# The pragmatics of exhaustivity in embedded questions: an experimental comparison of *know* and *predict* in German and English

**DOI:** 10.3389/fpsyg.2023.1148275

**Published:** 2023-09-13

**Authors:** Lea Fricke, Emilie Destruel, Malte Zimmermann, Edgar Onea

**Affiliations:** ^1^Department of German Studies, University of Bochum, Bochum, Germany; ^2^Department of French and Italian, University of Iowa, Iowa City, IA, United States; ^3^Department of Linguistics, Universität Potsdam, Potsdam, Germany; ^4^Department of German Studies, University of Graz, Graz, Austria

**Keywords:** experimental pragmatics, embedded questions, exhaustivity, probabilistic modeling, English, German

## Abstract

**Introduction:**

We present a cross-linguistic experimental study that explores the exhaustivity properties of questions embedded under wissen/to know and korrekt vorhersagen/to correctly predict in German and English. While past theoretical literature has held that such embedded questions should only be interpreted as strongly exhaustive (SE), recent experimental findings suggest an intermediate exhaustive (IE) interpretation is also available and plausible.

**Methods:**

Participants were confronted with a decision problem involving the different exhaustive readings and received a financial incentive based on their performance. We employed Bayesian analysis to create probabilistic models of participants' beliefs, linking their responses to readings based on utility maximization in simple decision problems.

**Results:**

For wissen/to know, we found that the SE reading was most probable in both languages, aligning with early theoretical literature. However, we also attested to the presence of IE readings. For korrekt vorhersagen in German, the IE reading was most probable, whereas for the English phrase "to correctly predict," a preference for the SE reading was observed.

**Discussion:**

This cross-linguistic variation correlates with independent corpus data, indicating that German vorhersagen and English to predict are not lexically equivalent. By including an explicit pragmatic component, our study complements previous work that has focused solely on the principled semantic availability of given readings.

## 1. Introduction

Although it is impossible to define the truth conditions of matrix questions because of their inquisitive nature, this problem is resolved once the question is embedded inside a matrix declarative sentence. So, for instance, while a question like (1) cannot be said to be true or false under any circumstances, it is possible to decide whether (2) as a whole is true or false by assessing whether Ali knows the correct answer to the matrix question.

(1) Who danced at the party?
(2) Ali knows who danced at the party.

To know the correct answer to a given matrix question comes down to understanding its answer conditions; in our example, this comes down to knowing how exhaustive (or complete) Ali's knowledge has to be for (2) to be true. Traditionally, the theoretical literature on question semantics distinguishes three levels of exhaustivity: strong, intermediate, and weak exhaustivity. These are explicitly spelled out in (3) for the embedded question in (2).^1^

(3) a. **Strongly exhaustive reading (SE)**
Ali knows/predicted for all people who danced at the party that they danced and he knows/predicted that they are the only ones who danced.
b. **Intermediate exhaustive reading (IE)**
Ali knows/predicted for all people who danced at the party that they danced and he does not have false beliefs/did not make a false prediction about any non-dancers.
c. **Weakly exhaustive reading (WE)**
Ali knows/predicted for all people who danced at the party that they danced.

The prevailing view in the theoretical literature throughout has been that the SE reading is the only available reading with *to know*, cf. Groenendijk and Stokhof (1984). This assessment was based on the reciprocal entailment of embedded questions and their negated counterparts as in (4)–the subsequent literature simply assumed this was the case; cf. Heim (1994) and Beck and Rullmann (1999).^2^

(4) Ali knows who danced ↔ Ali knows who did not dance.

Based on introspective judgments regarding the interpretative behavior of speech act verbs such as *to tell* or *to predict*, Klinedinst and Rothschild (2011) postulated the existence of IE-readings [cf. also Spector (2005, 2006), who argues for the existence of IE-readings also under *to know*, and Nicolae (2013), who uses the label WE for this reading, but who works with the false answer sensitivity typical of IE readings]. The empirical basis for assuming IE-readings over WE-readings with *to tell*/*to predict* is shown in example (5) (Klinedinst and Rothschild's, 2011, example [12], p. 7). Crucially, the final clause in the example is true in the context given: Arthur told us who sang because he provided the complete positive list of singers and was unsure about the rest (IE), whereas Bert did not tell us who sang because his list contained two false positives in addition to all the singers (WE). In general, such false positive beliefs are ruled out for the IE-interpretation.

(5) Context: Frank and Emilio sang.
A phone survey is taken to assess audience interest in last night's episode of a televised talent show by checking their recall of the contestants. Arthur says, “I'm sure Frank sang and Emilio sang, and I'm not sure about anyone else.” Bert says, “Frank, Emilio, Bill, and Ted sang.” It is decided to send a thank-you prize to Arthur but not Bert on the grounds that it is true that Arthur told us who sang, but Bert did not.

The same intuitive truth conditions are obtained for *to predict*, as also confirmed in subsequent experimental work by Cremers and Chemla (2016). In their experiment, the acceptance rate for the IE reading came close to the acceptance rate of the SE reading.

Table 1 provides an overview of the existing claims for the two verbs *to know* and *to predict* in the literature. Interestingly, different theoretical accounts make different predictions concerning the availability of SE/IE/WE readings for the two verbs. Summarizing the predictions for this issue therefore comes with difficulties, in part because the accounts under discussion have different (historical) backgrounds. In order to deal with this difficulty, the tables indicate whether an embedded question construction was found to be true (symbol ✓) in a particular SE/IE/WE-scenario (shown in each column) in the different works (* indicates that it was not found to be true). To illustrate, Karttunen (1977) assumes weak exhaustive question semantics. According to this view, the minimal requirement for a scenario to make (2) true is that Ali knows for all people who danced at the party that they danced. If Ali in addition has no false beliefs about the non-dancers (IE-scenario), or if he is aware that no other person danced (SE-scenario), (2) is also true. In contrast, the much stronger question semantics in Groenendijk and Stokhof (1984) only considers (2) true in a scenario corresponding to the SE-reading. Finally, notice that it is often unclear whether the judgments reported in the literature concern the semantic or pragmatic interpretation, for which reason we do not differentiate between the two layers of meaning.

**Table 1:** Literature overview: questions embedded under *to know* and *to predict*.

**Literature for “to know”**	**SE**	**IE**	**WE**
Karttunen (1977), Berman (1991) Sharvit (2002), Guerzoni and Sharvit (2007), and Spector and Egré (2007)	✓	✓	✓
Groenendijk and Stokhof (1984), Heim (1994), Beck and Rullmann (1999), Lahiri (2002), George (2011), Klinedinst and Rothschild (2011), and Theiler (2014)	✓	*	*
Spector (2006), Nicolae (2013), Spector and Egré (2015), Uegaki (2015), Theiler et al. (2018), and Zimmermann et al. (2022)	✓	✓	*
**Literature for “to predict”**	**SE**	**IE**	**WE**
Karttunen (1977), Berman (1991), Heim (1994), Beck and Rullmann (1999), Sharvit (2002), and Klinedinst and Rothschild (2011)	✓	✓	✓
Groenendijk and Stokhof (1984)	✓	*	*
Spector (2006), Theiler (2014), Spector and Egré (2015), Uegaki (2015), and Theiler et al. (2018)	✓	✓	*

Another line of research on (embedded) questions takes psychological aspects of the communicative situation the question occurs in into account. Ginzburg (1995a,b) suggest a situation-theoretic analysis of questions and discuss questions in terms of resolvedness. Whether an answer resolves a question is dependent on the goal and the knowledge state of the questioner. Crucially, an answer does not necessarily need to be exhaustive in order to resolve a question. Van Rooij (2004) presents a decision-theoretic approach to questions. According to this theory, preference for an exhaustive or a non-exhaustive answer depends on its utility in a given context. Asher and Lascarides (1998) present an SDRT approach, which incorporates besides semantic aspects the discourse context and the cognitive states of the interlocutors. Based on the observation that a number of *wh*-questions, particularly those involving the *wh*-words *how* and *why*, do not require exhaustive answers, Asher and Lascarides (1998) analyze exhaustiveness as a pragmatic effect. Similarly, in the approach by Schulz and van Rooij (2006) the exhaustive interpretation is analyzed as a Gricean implicature which is dependent on relevance in the respective context.

The previous experimental work on embedded questions has, however, not incorporated pragmatic aspects so far. Some of the results of the experimental research on this topic are at odds with the introspection-based judgments from the semantically-oriented theoretical literature: For instance, the acceptance rate of IE readings for questions embedded under English *to know* (Cremers and Chemla, 2016) and French *savoir* (Cremers et al., 2017) was found to be around 90%, which was similar to the acceptance rate of SE-readings in those experiments. At the same time, and contrasting with this positive evidence in support of the availability of IE readings with embedded questions, Cremers and Chemla (2017) found no evidence for the availability of IE readings with embedded questions in another experimental study on English *to know*.^3^

The experiments in Cremers and Chemla (2016) and Cremers et al. (2017) all used truth-value judgment tasks with picture verification. In Cremers and Chemla (2016), experiment 2, the context and mental state of the attitude holder were given in the form of pictures, which, as [anonymized] commented, allows for participants to use a low-level response strategy: they could only compare the pictures to check whether the positive answer space is aligned, thereby ignoring the uncertainties in an IE situation. However, in Cremers et al. (2017) such a low-level response does not seem likely, as the mental state of the attitude holder was presented in the form of a statement. Still, this experiment yielded equally high acceptance for IE readings.

The experimental studies mentioned aimed at testing for all readings that are acceptable for embedded questions one way or another. However, it may not be entirely clear what it entails if a reading is accepted in an abstract experimental setting, such as a picture-verification task. Would the participants themselves actively use the target sentence on the relevant interpretation in the respective context? Or would they merely consider it possible that somebody else might understand the sentence in this way, possibly based on a more liberal use of the language? In short, experimental settings in which nothing is at stake for the participants open up the possibility that lay participants liberally accept interpretations that they themselves would not consider optimal and which they themselves would therefore never actively employ in a communicative situation. In fact, there may be various reasons for participants to accept an interpretation in a given experimental setting, some of them non-linguistic, such as, e.g., agreement with the content of the stimulus. There may be, hence, a difference between the mere availability of a reading in non-communicative experimental tasks and the active employment of a reading of an embedded question in practical communication as an active part of a speaker's linguistic repertoire. What all this amounts to is that, even if a reading is accepted in an experiment, this does not necessarily mean that the availability of this reading is relevant from a pragmatic communicative perspective; see also Franke (2016) on the importance of linking functions between experimental results and theoretical assumptions in experimental pragmatics.

A number of studies in experimental semantics and pragmatics have shown that the methodological features of an experiment can dramatically affect its outcome. Take the debate around the existence of scalar implicatures in embedded contexts, for instance. Geurts and Pouscoulous (2009) showed that participants derive embedded scalar implicatures more frequently in inference tasks than in truth-value judgment tasks. Likewise, Benz and Gotzner (2014) point out that the reason for a low rate of implicatures in an experiment can be due to an experimental design that favors semantic interpretations for the simple reason that pragmatic inferences are of no relevance in the context of the experiment. The role of the context is also emphasized by Zondervan (2010), who investigated the role of information focus on the generation of scalar implicatures. Similarly, a study by Degen and Goodman (2014) demonstrated the impact of QUD and set size on the generation of scalar implicatures as well as task-specific effects.^4^ In order to come closer to natural language use than classic experimental set-ups, Benz and Gotzner (2021) employed an interactive game-theoretic design which included a speaker, a listener, and a communicative goal.

The experimental studies we present in this paper head in a similar direction. Instead of investigating the general availability of different exhaustivity readings for embedded questions, we approach the issue by investigating which readings speakers actually commit to in a decision task. Our experimental design aims at removing the postulated charity-based tendency in participants for allowing a wider range of interpretations than they would actually use themselves. It complements existing experimental approaches in setting up a more practical pragmatic context, which in turn allows for the probabilistic modeling of individual participants beliefs. The main features of our experiment are as follows:

(1) Participants get a financial incentive based on their individual performance during a decision problem that takes the form of a betting scenario involving the three readings SE, IE, and WE. The incentive had two important aspects: First, it aimed to increase the overall performance level in terms of attentiveness and motivation; second, because the conscious choice of one reading over another has direct financial consequences, it allows for the probabilistic modeling of participants' belief contents.
(2) The experimental design complements previous approaches, which focused on the mere availability of a given reading, with an explicit pragmatic component. In particular, the advertised financial gain will motivate participants to engage in active pragmatic reasoning over their own and other speakers' interpretations of embedded questions. An important factor for solving the decision problem in the betting scenario is the question of whether the participants' interpretations match that of other members of the linguistic community or not.
(3) In addition, we engaged in a cross-linguistic comparison of English questions embedded under *to know* and *to correctly predict* on the one hand and their German counterparts under *wissen* and *korrekt vorhersagen* on the other. The cross-linguistic comparison within the same pragmatic setting also serves two purposes. First, it helps us validate the experimental method, as we would expect overall comparable results for the two languages. More importantly, however, such a cross-linguistic comparison may help us unearth possibly subtle lexical differences in the embedding predicates, which in turn would be reflected in slightly different interpretive behaviors of speakers of the two languages.

The linking hypothesis of our experiment is based on utility theory for simple decision problems: Participants aim to maximize their expected utility, measured in terms of financial payoff.^5^

## 2. Experiments

Our experimental design addresses two common problems with (linguistic) experiments. First, experiments present participants with a cognitively demanding task. Participants have to concentrate for a (considerable) length of time and must generate interpretation-based judgments based on numerous stimuli that are often quite similar. Lack of motivation and/or increasing fatigue may lead to superficial reading and shallow processing of the experimental stimuli and, at worst, may result in the selection of a random answer. This type of behavior is described by Krosnick (1991, 1999), who subsumes it under the label *satisficing*, a notion originally coined by Simon (1957). According to Vanette and Krosnick (2014) “when faced with [...] demanding information-processing tasks[...][,] people often expend only the amount of effort necessary to make an acceptable or satisfactory decision” (see also Kool et al., 2010 on avoidance of cognitive demand). In particular, such experimental settings in which the participant has to click through a repetitive experiment while seated alone in front of a computer seem prone to the induction of satisficing strategies. It seems that often compensation for mere participation is an insufficient incentive for participants to attempt to come up with the best response in each and every experimental trial.

Second, as already discussed above, it is often not clear what can be concluded from judgments in linguistic experiments which test the general availability of an interpretation without controlling for the status of that interpretation. When judging a statement, one could judge it either based solely on its semantic/logical interpretation or based on the interpretation that one expects will most likely be shared by other speakers and therefore is of communicative relevance. In other words, is a reading merely accepted on a charitable interpretation or is it accepted because it is the intended interpretation in a communicative situation? (This is not to say this must necessarily be a pragmatically enriched interpretation – a semantic interpretation can be of communicative relevance, too.).

In view of these methodological considerations, we aimed to design an experiment^6^ that maximizes rational behavior in participants and makes them want to find the optimal response in each and every experimental trial. We consider an interpretation optimal not only if it is the best solution arrived at after intensive thinking but also if it is an interpretation that can be expected to be shared by other speakers. To ensure that participants would engage in recursive thinking of this kind when responding to the trial stimuli they were told that they would lose money if another person does not agree with their judgment. Thus, every individual response from a participant had direct financial consequences for them.^7^ This way, it was in participants' own interest to think carefully about each individual item.

Moreover, we aimed to create a diverse and entertaining item set. For the German version of the experiment, we also chose an interactive lab setting in order to prevent fatigue in participants and in order to increase their commitment. It is important to note that we were unable to have the exact same setting for the English version of the experiment—face-to-face interactions were prohibited due to restrictions linked to the COVID-19 pandemic. We will keep in mind these differences, however, when interpreting the results.

### 2.1. Experiment 1: German

In this experiment, we tested the interpretation of embedded *wh*-questions under the German verbs: *wissen* “to know” and *korrekt vorhersagen* “to correctly predict.” The verb *vorhersagen* on its own can be interpreted to mean “to make a prediction” or to mean “to correctly predict.” As the latter interpretation was intended, we chose the form *korrekt vorhersagen* “to correctly predict.”

#### 2.1.1. Participants

In total, 24 native speakers of German (mostly Austrian German) were tested, 17 females and seven males, who were between 20 and 31 years old (M = 24.37 years); 20 of them were University students, five were employees, one did not answer this question. They were recruited via postings on university-related Facebook groups and via printed posters on campus. The financial compensation varied between 9.40 and 10.40 euros.

#### 2.1.2. Materials

In the experiment, participants had to judge test items in the context of role play. They were presented with the context in (6).

(6) In a reality show called *The Glass House*, the five contestants, Alessa, Carlo, Freddy, Mara, and Sophie, are filmed during their activities in the house and while doing certain things for a dare. In a special episode at the end of the season, the presenters, Tim and Tiffany, are looking back at the season. In doing so, they have to answer questions about the contestants and their activities in the house. Beforehand, viewers could place bets on what Tim and Tiffany know.

**Figure 1:**
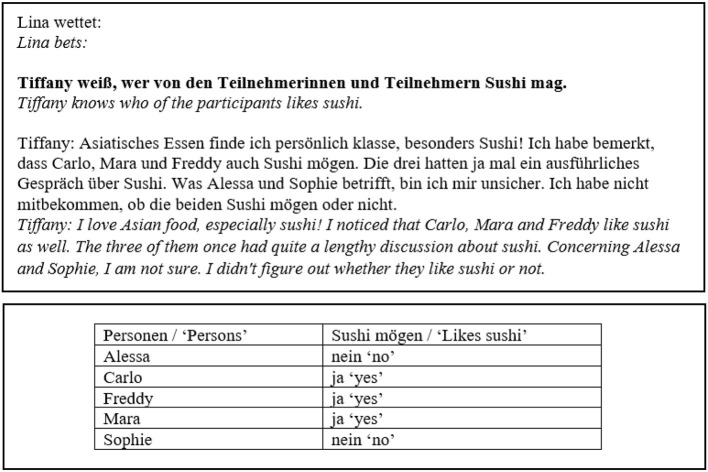
Betting slip, *wissen*, IE, -neg.

**Figure 2:**
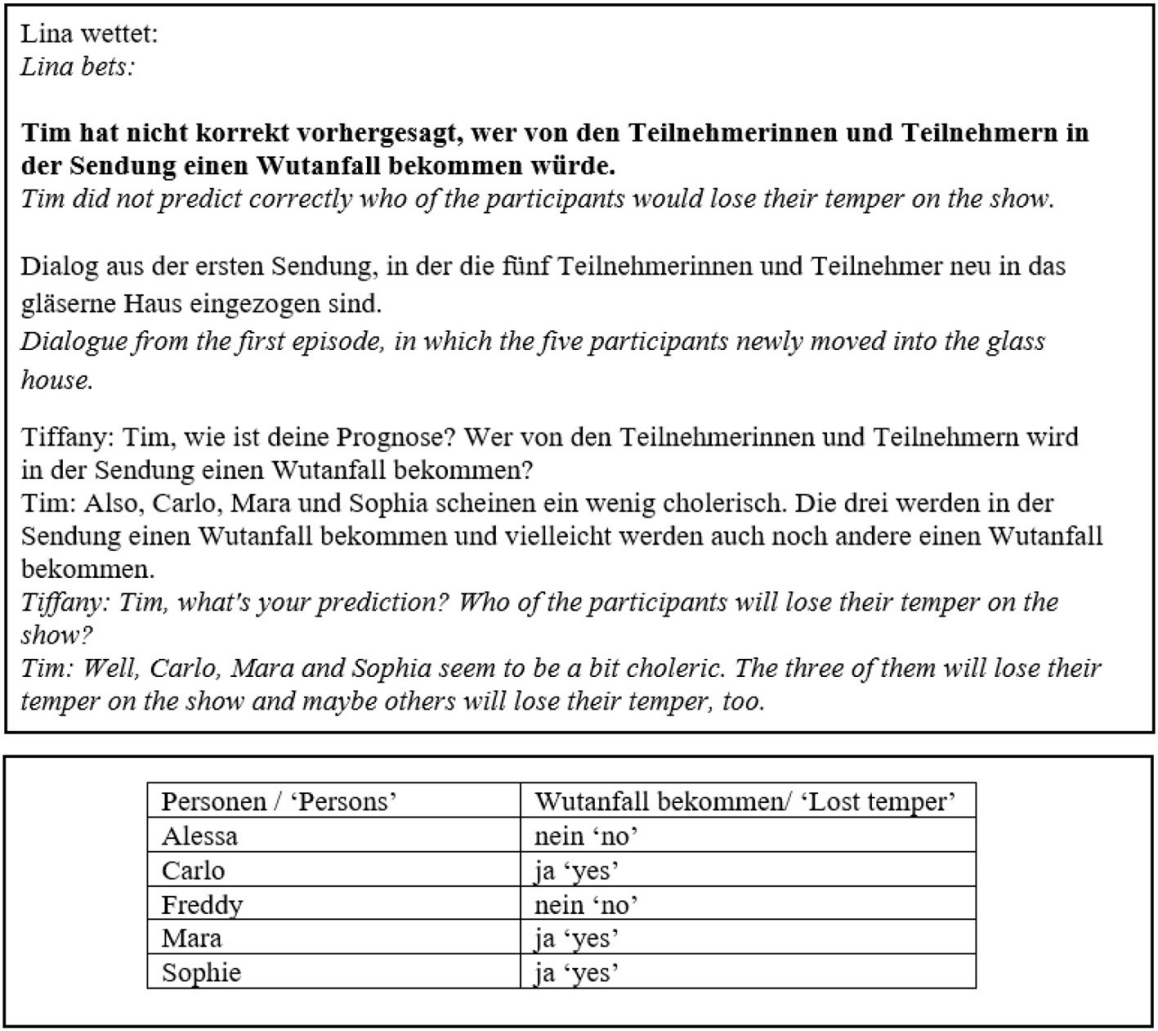
Betting slip, *korrekt vorhersagen*, IE, +neg.

The concrete experimental task was for participants to evaluate the outcome of the bets, so the experimental stimuli were presented on betting slips. Examples for betting slips are shown in Figures 1, 2. Judging a bet as won corresponds to accepting a target sentence. The betting slip had two sides. The front side included the bet in form of (i) a question embedded under one of two matrix verbs and (ii) a monolog or dialogue in which the beliefs of the attitude holder in question were expressed, see also example (7) for an illustration of the general structure of the betting slips. The two matrix verbs we used to embed the questions were *wissen* “to know” and *korrekt vorhersagen* “to correctly predict.”^8^

(7) *Frontside*
**Lina bets: X** **<+/- negation>** **< verb>** **< Q>**
Dialogue/Monolog: Contextual information that X < attitude> that < answers to Q>
*Backside*
< Facts in the world>

The backside of the slip showed a table displaying what had actually happened, i.e., facts in the world. This mode of presentation was chosen to prevent participants from using a low-level answering strategy,^9^ i.e., they could not simply look for differences between two sentences printed on the same page. The contents of the monolog/dialogue and the reported actual facts in the world were manipulated in the template to yield the different readings by changing the values of the variables *attitude, answers to Q* and *facts in the world*, as illustrated in Table 2. Reading constitutes the first factor in our experiment.

**Table 2:** Readings tested in Experiment 1 (A+ = A has Q-property, A− = A does not have Q-property, A? = uncertain whether A has Q-property).

**Reading**	**Statement of attitude holder**	**Facts in the world**
SE	A+, B+, C+, D−, E−	A+, B+, C+, D−, E−
IE	A+, B+, C+, D?, E?	A+, B+, C+, D−, E−
WE	A+, B+, C+, D+, E−	A+, B+, C−, D−, E−

The second factor, negation, was tested within participants and between items. As indicated in (7), the embedding matrix predicate was either negated or not. We included this factor to see whether participants' judgments were consistent. To illustrate, if participants were consistent in their judgments, they should accept the target sentence in the condition SE_−neg_ shown in (8-a), that is judge the bet as won, and reject the target sentence in the condition SE_+neg_ shown in (8-b), which means judging the bet as lost, and vice versa. However, because of homogeneity issues with negated items, we have eventually excluded the negated data from the final data analysis.

(8) a. **−neg** Tiffany knows who of the participants wears contact lenses.
b. **+neg** Tiffany does not know who of the participants wears contact lenses.

As Figures 1, 2 show, there was a difference in the wording between the IE conditions of the two verbs. While in the case of *wissen*, the two candidates the attitude holder was unsure about were named explicitly, the wording in the case of *korrekt vorhersagen* was more vague (“and maybe others might do × as well”). We will return to this matter later in the discussion section.

In addition to the item manipulations, we included a third factor at the participant level, i.e., role. This factor was tested between subjects and within items. It was used to control for an answering bias, i.e., a tendency to judge bets won (or lost) in order to maximize profit. Participants took on either role 1 or role 2 in their interaction with the experimenter, so half of the participants acted in role 1 and the other half in role 2. In role 1, participants had to decide whether or not to redeem the bet at a betting office. They were told that their friend, Lina, had placed some bets but did not have time to go to the betting office to cash in her bets. Instead, the participants were asked to go on her behalf, for which they would earn a share of the profits. They received 5 euros of starter cash. Redeeming a bet cost a fee of 10 cents each. For each redeemed bet that was actually won the participants received 30 cents in return. Thus, in effect, the participants gained 20 cents for a redeemed bet which was won, and they lost 10 cents for a bet that was lost. In this role 1, the participants profit financially from bets that are won, for which reason they may be biased toward judging difficult borderline cases as won bets. However, this bias is harnessed in by the fee for submission, as the participants will lose money by randomly submitting bets. Thus, the participant had to take into account that an authority that decides about the status of the bets would share their judgment.

In role 2, participants acted as the clerk in the betting office. They had to decide for each redeemed bet whether it was won or not. Participants received 15 euros of starter cash. For a won bet, they had to pay out 20 cents. If they decided incorrectly that a bet was lost which was actually won, there was a deduction of 30 cents. Participants were told that Lina would raise an objection if she did not receive her gains from won bets. Thus, they had to consider whether their interpretation would be shared by Lina. To sum it up, in role 2 participants profit from lost bets, for which reason they could be biased toward not paying out bets, but the financial deduction for incorrect decisions served to harness the bias.

Note that in calculating the compensation a participant received, decisions on test items were always considered correct. However, as there was a balance of non-negated and negated target sentences in each condition this did not mean that a person in role 1 who interpreted questions as strongly exhaustive gained less money than a person with a weakly exhaustive interpretation. The fact that a participant accepted one exhaustive interpretation and rejected the other did not affect their final compensation provided that they answered in a consistent manner. In the case of fillers, as explicated below, there were, in fact, correct and incorrect answers. Thus, making mistakes on fillers negatively affected the final compensation.

The factorial design was 3 (reading) × 2 (negation) × 2 (role) for each of the two embedding verbs. The first factor reading had three levels, i.e., SE, IE, and WE. This factor was tested within subjects and within items (per verb). The second factor, negation, was tested within subjects and within items, and the third factor, role, was tested between subjects and within items.

For each verb, we created a set of six lexicalizations, yielding a total of 12 test items. In addition to these test items, we created 26 fillers that also served as controls. The fillers also involved questions embedded under the verbs tested in the experiment. They were not only constituent questions like the target items but were of various types (e.g., adjunct questions and polar questions).

The experimental items were distributed over six experimental lists with four participants per list. The combination of the six conditions [three readings × (+/-) negation] and six lexicalizations varied systematically within each verb between lists. That is, one lexicalization occurred in a different condition on each list.

#### 2.1.3. Procedure

The experiment was conducted in a lab setting. Before the start of the experiment, participants were asked for their demographic data. Depending on the role they were assigned, they received 5 or 15 Euro of starter cash in stacks of 10 and 20 cent coins. The experimenter handed the betting slips one by one and in randomized order to the participant, who then had to decide whether to redeem the betting slip (role 1) or pay out the gains (role 2). If they wanted to redeem or pay out, depending on the role, they had to return the betting slip together with the money to the experimenter. The experimenter entered the participant's decision into an Excel sheet that automatically calculated the sum the participant received as financial compensation after the experiment. The participant did not receive any feedback as to his/her gains and losses from individual bets, neither during nor after the experiment. As a warm-up phase, participants saw three trial items before the start of the actual experiment. They served to get the participants accustomed to the task. After processing the first half of the betting slips there was a short break. The experiment took 25–40 min in total.

#### 2.1.4. Results

##### 2.1.4.1. Descriptive results

No data were excluded from the analysis. For each verb, we collected 24 data points per condition. Table 3 shows the acceptance rates per condition for both verbs in percentage and absolute numbers. The SE_noneg_ reading receives at-ceiling, the IE_noneg_ reading medium and the WE_noneg_ reading no acceptance. For *korrekt vorhersagen*, the resulting pattern in the SE and in the WE conditions is like the one of *wissen*. In contrast, we see higher acceptance for the IE_noneg_ reading than for *wissen*. For both verbs, the results in the negated and non-negated conditions roughly match up. These descriptive data indicate no striking differences between the two roles.

**Table 3:** Acceptance in percent by role for the two verbs (absolute numbers in brackets).

**Wissen**		
**Condition**	**Role 1**	**Role 2**
SE no neg	100% (12)	100% (12)
SE neg	8% (1)	0%
IE no neg	42% (5)	50% (6)
IE neg	67% (8)	50% (6)
WE no neg	0%	8% (1)
WE neg	100% (12)	92% (11)
**Korrekt vorhersagen**		
**Condition**	**Role 1**	**Role 2**
SE no neg	100% (12)	100% (12)
SE neg	8% (1)	0%
IE no neg	100% (12)	83% (10)
IE neg	8% (1)	8% (1)
WE no neg	0%	0%
WE neg	92% (11)	92% (11)

##### 2.1.4.2. Inferential statistics

Even though it is obvious by inspecting the raw data already that the factor *role* did not play a role in the decision making, to test for an effect of the factor role we fitted Bayesian generalized linear mixed models using the software R (R Core Team, 2022) and the package rstanarm (Goodrich et al., 2022). A Bayesian approach has the advantage that, in contrast to frequentist models, it can easily deal with conditions in which there is no variance as is the case in the SE and WE conditions. For each verb, we created a model, which included a factor role and a model which did not include a role as a factor [see (9)]. We used default priors, which are weakly informative.

(9) a. stan glmer[response ~ reading * negation + role + (1|participant) + (1|item), family = binomial, iter = 45000]
b. stan glmer[response ~ reading * negation + (1|participant) + (1|item), family = binomial, iter = 45000]

Model comparison using Bayes Factors (Makowski et al., 2019) yielded substantial evidence that the model which did not include the factor role was superior (*wissen*: BF = 6.14, *korrekt vorhersagen*: BF = 3.61).

#### 2.1.5. Discussion

We postpone a comprehensive discussion of the results, which takes into account Bayesian data analysis to Section 4 and note only a few observations here.

For *wissen*, the complete acceptance of the SE reading and the marginal acceptance of the WE readings are both in line with judgments in the theoretical literature and with the previous empirical literature. However, the IE reading received considerably less acceptance than in the experiments in Cremers and Chemla (2016) (English) and Cremers et al. (2017) (French). Moreover, we note that the results of this experiment do not indicate a bias stemming from the experimental factor role (redeeming vs. paying out bets).

By contrast, the high acceptance of the IE reading of *korrekt vorhersagen* is in line with the judgments in the more recent literature and the experimental data in Cremers and Chemla (2016).

### 2.2. Experiment 2: English

Experiment 2 is the English version of Experiment 1 and tested English *to know* and *to correctly predict*. However, as described below, there are a few small differences as compared to Experiment 1 regarding materials and procedure.

#### 2.2.1. Participants

For the English experiment, we tested 26 monolingual native speakers of American English: 12 males and 14 females who were aged between 19 and 24 years old (M = 20.65 years). All were either undergraduates (n = 20) or graduate students (n = 6) in a Midwestern university and were recruited via email messages. All students who participated, undergraduate and graduate students alike, received extra points (in %) toward their final homework grades in a given course (a language course for the undergraduates and a literature seminar for the graduates). The number of points received corresponded to the amount of gain in the study (varied between 8.80 and 11.40 dollars).

#### 2.2.2. Materials

The materials were the same as in Experiment 1 with three differences. (1) We adapted the names of the characters in the story for an English-speaking audience. (2) The fillers in this experiment were partly the same as before and partly test items of another experiment investigating scalar implicatures. (3) The wording of the testitem for *to correctly predict* in the IE condition differed slightly. As shown in (10), instead of saying “and maybe others will do × as well,” the attitude holder names those candidates they are uncertain about explicitly. We made this change to make the presentation of IE conditions parallel to the IE conditions of *to know*, in which the candidates the attitude holder is uncertain about are also named explicitly. We return to the possible effects of the second two differences between the experiments in the discussion section.

(10) *To correctly predict, IE, +neg*
**Lina bets: Tim didn't correctly predict who of the participants would throw a tantrum on the show**.
Dialogue from the first episode, in which the five participants had newly moved into the glass house.
Tiffany: Tim, what's your prediction? Who of the participants will throw a tantrum on the show?
Tim: Well, Carlos, Mary, and Alicia seem to be a bit choleric. The three of them will throw a tantrum on the show, and maybe Sophia and Freddy might throw a tantrum as well.

#### 2.2.3. Procedure

The experiment took place online due to restrictions placed by the COVID-19 pandemic on collecting data in person. The experiment was adapted to fit an online format, thus there was no experimenter physically distributing betting slips, rather the experiment took place in a video call and participants completed the task in front of their personal computers. Upon recruitment (via email), when subjects accepted to take part in the experiment, they received a PDF document that included the instructions for the role they were assigned. The last difference with the German version was that, instead of receiving actual money, participants saw pictures representing their gains/losses, and were given point credit toward their final homework grades. The experiment included a trial session with three items, after which the participants saw a message displaying “The experiment starts now.” Participants got a “break” screen after half of the items. Overall, the experiment took about 25–30 min to complete. Personal data were collected at the end of the task.

#### 2.2.4. Results

##### 2.2.4.1. Descriptive results

The data of 1 participant was excluded based on having made more than three mistakes on filler items. The data from the remaining 25 participants were analyzed. We collected 25 data points per condition. Table 4 compares for each verb the acceptance rate for each condition by role.

**Table 4:** Acceptance in percent by role for the two verbs (absolute numbers in brackets).

**To know**		
**Condition**	**Role 1**	**Role 2**
SE no neg	100% (13)	100% (12)
SE neg	0%	8% (1)
IE no neg	46% (6)	50% (6)
IE neg	69% (9)	67% (8)
WE no neg	12% (2)	8% (1)
WE neg	62% (8)	92% (11)
**To correctly predict**		
**Condition**	**Role 1**	**Role 2**
SE no neg	100% (13)	100% (12)
SE neg	8% (1)	0
IE no neg	31% (4)	58% (7)
IE neg	38% (5)	25% (3)
WE no neg	0%	0%
WE neg	92% (12)	100% (12)

For *to know*, we find at-ceiling acceptance for the SE_noneg_ reading, acceptance at 48% for the IE_noneg_ reading, and low acceptance for the WE_noneg_ reading. The acceptance rates for non-negated and negated conditions match up only for the SE reading. For *to correctly predict*, the SE_noneg_ reading received at-ceiling acceptance while the WE_noneg_ reading was rejected. For these readings, the results of non-negated and negated conditions are complementary. The IE_noneg_ condition received 44% acceptance while IE_neg_ only received 32% acceptance, which is not complementary.

##### 2.2.4.2. Inferential statistics

To test for an effect of the factor role, we create two Bayesian generalized linear mixed models for *to know* and *to correctly predict*. Following the procedure of Experiment 1, one model included role as a factor while the other did not. Model comparison using Bayes Factors (Makowski et al., 2019) yielded for *to know* strong evidence that the model which did not include the factor role was superior (BF = 10.76) and substantial evidence in the case of *to correctly predict* (BF = 7.84).

#### 2.2.5. Discussion

For *to know*, the results in the SE and IE condition, have the same pattern as for German *wissen*. However, there is slightly higher acceptance for WE_noneg_ and a lower acceptance for WE_neg_ than in the German experiments. For *to correctly predict*, the results in the SE and WE conditions show the same pattern as German *korrekt vorhersagen*. The IE condition, in contrast, has a lower acceptance rate in English.

## 3. Bayesian modeling

In this section, we develop a statistical model for the experimental data with a linking hypothesis between participants responses and readings, which is based on utility maximization in simple decision problems. The type of problem is usually known as a decision under uncertainty (Knight, 1921; Savage, 1954). Decision theory has been applied to exhaustivity of questions already in the work of Van Rooij (2004). Moreover, Hawkins et al. (2015) have developed a Rational Speech Act model for questioning and answering.

In our model, we assume that the preferences are perfectly matched by the payoff, i.e., participants wishing to maximize their income, so that the subjective expected utilities can be exactly calculated on the basis of the subjective probabilities for the different readings together with the expected financial payoffs for the various outcomes. Of course, one could also conceive of this decision problem as a basic game played by a participant against nature (see Jaeger, 2008 or Franke, 2013 on the use of game theory in linguistic research).

The first elements we need for our analysis are the raw financial payoff tables of the two roles in Table 5. Decisions are marked on the vertical axes, and the states of the world on the horizontal axes. Hence, the top left cell represents the net payoff of 20 cents for a bet that is indeed won if the decision is to redeem it; by contrast, the bottom left cell represents a net loss of 10 cents, if the decision is to redeem a bet which is in fact lost. The table also introduces the terminology that we will be using in the rest of this section in boldface, thus glossing over the differences between the two roles. In particular, we will refer to the case in which the betting slip is winning as the *true* case and to both redeeming and paying out the bet as *action*. For convenience, we further summarize in Table 6, as already discussed above, under which readings a bet would be considered true or false in each condition/state.

**Table 5:** Payoff table for role 1 and role 2.

**Role 1**		
	**Action (redeem bet)**	**No action (not redeem bet)**
True (Bet won)	20	0
False (Bet lost)	−10	0
**Role 2**		
	**Action (redeem bet)**	**No action (not redeem bet)**
True (Bet won)	20	−30
False (Bet lost)	−20	0

**Table 6:** Readings verifying each condition.

	**State SE**	**State IE**	**State WE**
True	Reading: SE, IE, WE	Reading: IE, WE	Reading: WE
False	Reading: ∅	Reading: SE	Reading: IE, SE

The optimal strategy for participants under the current assumptions is very clear: choose action or no action depending on which one has the higher payoff in the given situation, i.e., whether the bet is true or false. However, as is generally the case in decision problems under uncertainty, we assume that participants may be uncertain about (some of) the readings. After all, both introspection data and the literature survey have made sufficiently clear that people are often not 100% sure about whether a specific reading obtains or not. We can therefore compute subjective expected utilities given uncertainties about the readings (remember that the states are fixed!). We can do this in the standard way as shown in (11).^10^ Then, the optimal strategy is simply to choose the action if the expected utility of the action is higher than the expected utility of no action.

(11) Standard utility function:

$$\begin{array}{l} U\left(action|state\right)=\underset{reading}{\sum }\text{Payoff}\left(state,reading,action\right)*p\left(reading\right)\\ U\left(noaction|state\right)=\underset{reading}{\sum }\text{Payoff}\left(state,reading,noaction\right)\\ \;*\;p\left(reading\right) \end{array}$$

With this in mind, we have implemented a hierarchical statistical model in STAN using the RStan package in R (R Core Team, 2022; Stan Development Team, 2022). Thereby, we modeled the decision made by individuals as a *bernouli*_*logit* decision with the computed utility of action as a sole parameter.

**Figure 3:**
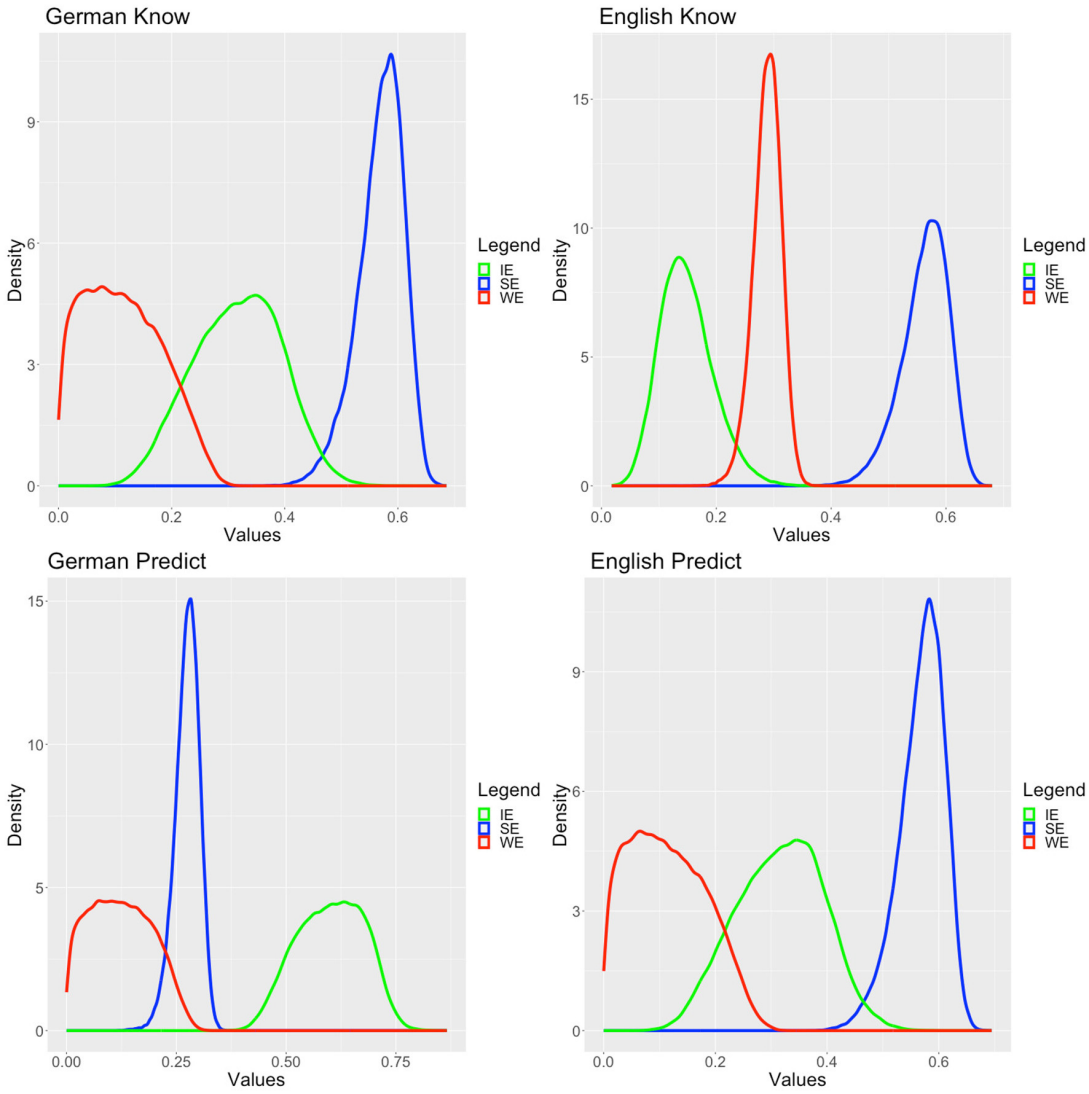
Standard model posteriors.

We have used informative but not very strong priors based on the literature. In particular, we have assumed that SE and IE are more likely than WE, as depicted in Figure 3 and in equation (12). While we were aware that in the literature discrepancies between *to predict* and *to know* were discussed, given that our priors are rather flat, we wished to remain fairly uncommitted and therefore choose to keep the same priors for both verbs and both languages.

(12) Priors:

[SE,IE,WE]~dirichlet(2,2,1.2)

The resulting model, which we dub the *standard model*, is outlined in (13).

(13) Standard Model

$$\begin{array}{l} \left[SE,IE,WE]\sim \text{dirichlet}(2,2,1.2\right)\\ y[i]\sim \text{bernoulli}\_\text{logit}(Utility(state[\text{i}],\\ \left[SE,IE,WE])\right) \end{array}$$

**Figure 4:**
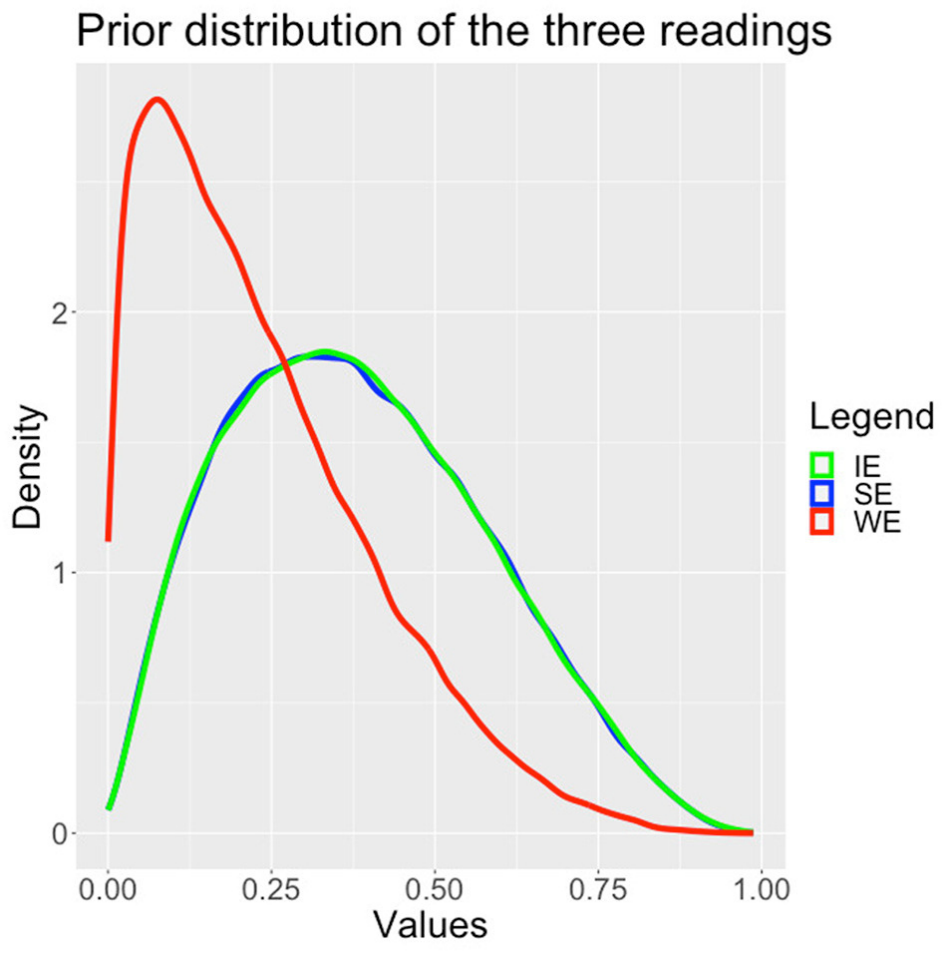
Priors.

We ran the model using a Hamiltonian Monte Carlo simulation with the no-U-turn sampling (NUTS) algorithm (Hoffman and Gelman, 2014) with 20,000 iterations and 10,000 warmups on eight parallel chains for each verb/language. In order to avoid a very small number of divergent transitions after warmup diagnosed by Rstan, we reduced the stepsize by setting the parameter adapt_delta to 0.95. All simulations had excellent convergence both based on usual visual diagnostics and Rhat values (Gelman and Rubin, 1992; Vehtari et al., 2021). The latter are provided in the Supplementary material. The posteriors are plotted in Figure 4.

To check the quality of the predicted models, we conducted the usual posterior predictive checks and observed good fit with the data (see Supplementary material). ^11^

Of course, however, the standard model has the built-in drawback that it assumed that all participants have the same probabilistic distribution for the three readings. However, it might also be the case that individuals are subject to some variation. To attack this possibility, we constructed an alternative model that was meant to improve on the standard model. In this model, we assume that the probabilities that individuals have for the three readings are distinct, i.e., different individuals have different probabilities for the three readings. For example, if some of the readings are computed pragmatically based on contextual factors and using potentially distinct strategies, individuals could in fact arrive at relatively different subjective probabilities. We dub this model a variable-value model. The model is described in (14).

(14) Variable value model

$$\begin{array}{l} \sigma \sim \text{beta}(1,20)\\ \text{for }i\text{ in }(1:N\_persons):\{\\ \left[SE[i],IE[i],WE[i]]\sim \text{dirichlet}(2,2,1.2\right)\\ \text{for }j\text{ in }(1:N\_trials):\{\\ y[i,j]\sim \text{bernoulli}\_\text{logit}(Utility(state[j],[SE[i],IE[i],\\ WE[i]]))\}\} \end{array}$$

**Figure 5:**
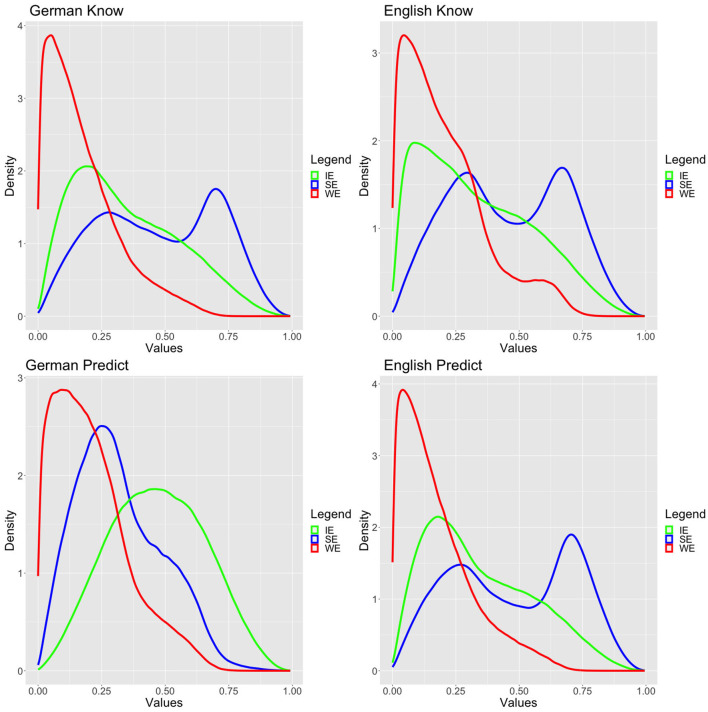
Variable value model posteriors.

All simulations converged by achieving excellent Rhat values, and they passed the usual visual diagnostics, but since the readings were simulated for each participant individually, we refrain from presenting a large table with Rhat values. A reduction of stepsize was not necessary. The posteriors of the variable value models are presented in Figure 5.

We have performed model comparison using the Bayes factor (BF) with the bridgesampling package (Gronau et al., 2020). In particular, we compared samples with a total of 50.000 iterations and a warm-up of 10.000 for both models. We present the model comparison for each of the data sets in the Table 7.

**Table 7:** Bayes factor model comparison.

**Data set**	**BF (variable value model | standard model)**
German wissen	7.508e+16
English know	3.438e+18
German korrekt vorhersagen	9.2e−4
English correctly predict	1.862e+20

There is a clear difference between German *korrekt vorhersagen* and all the other data. In particular, for German *korrekt vorhersagen*, while the variable value model achieved excellent fit, it is clearly inferior to the standard model. More importantly, as can be visually confirmed in Figure 5, the three readings' probabilities are more or less normally or lognormally distributed. This suggests that there are no groups or dialects, which would apply varying strategies for dealing with the experimental task for German *korrekt vorhersagen*. This perfectly fits with the Bayes factor diagnostic that the best model is the standard model, which assumes precisely good agreement in the population about the readings, i.e., the dominant reading is the IE reading. For all the other cases, the variable value model is incommensurably more appropriate than the standard model. This we take as clear evidence—at least based on our data—that there cannot be any population-level stable distribution of the three readings for these verbs. Indeed, one can also visually confirm this by inspecting the posteriors, which exhibit several peaks and are far away from normal or normal-like distributions resembling more bimodal distributions.^12^

**Figure 6:**
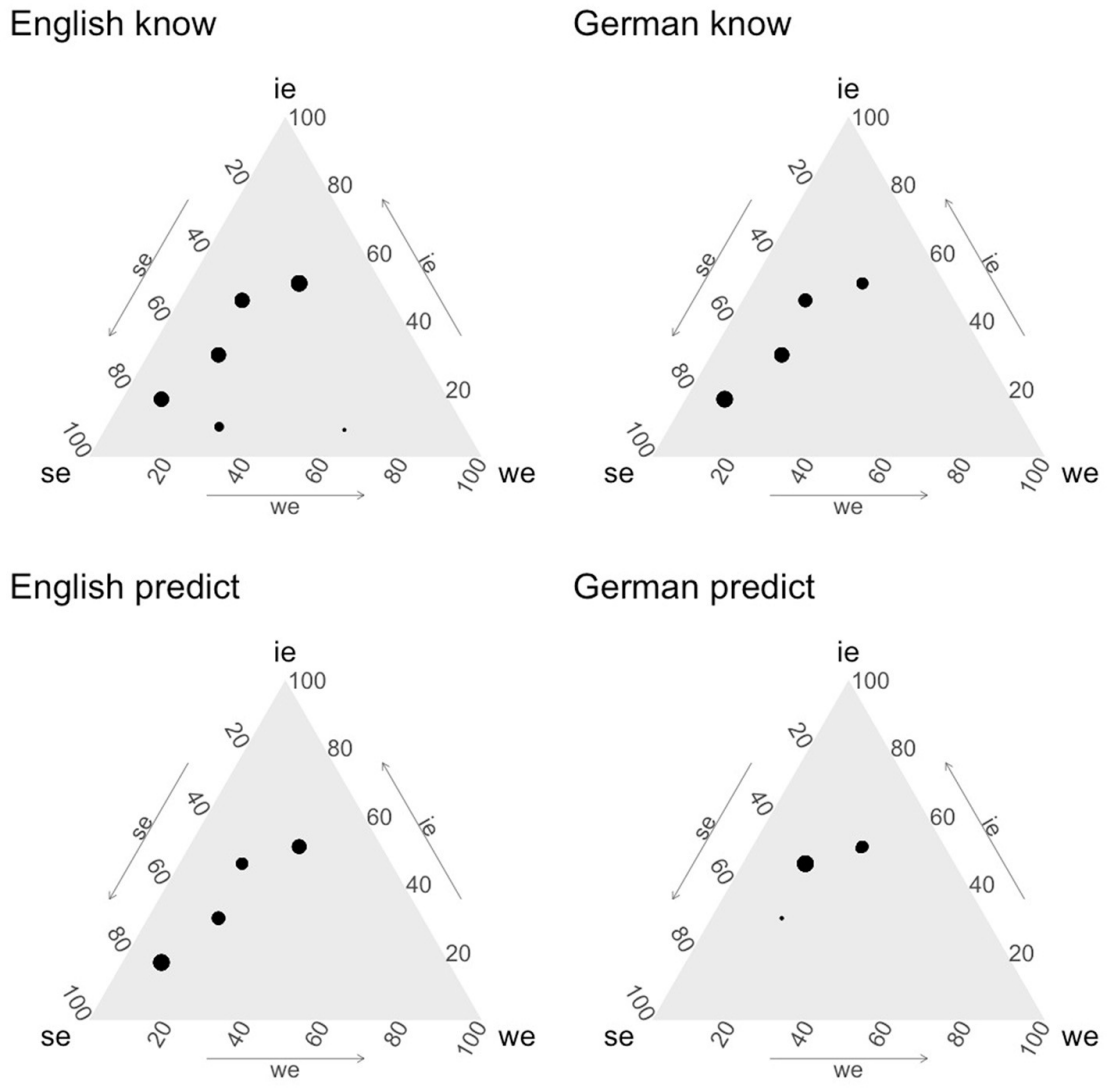
Variable value model mean posteriors.

Since we have reason to believe that there are different types of speakers in the population for at least three of our language/verb combinations, we need to get a more detailed picture of the types of individuals that can be found. Accordingly, in order to get a better diagnosis of how to interpret the variable value models for the two verbs and languages, we plotted ternary plots using the package ggtern (Hamilton and Ferry, 2018). In these plots, each dot represents a type of participant according to the mean simulated SE, IE, and WE probability, and the size of the point represents the frequency of that type of participant in the simulated data. The results are shown in Figure 6.

From these plots we can cautiously conclude that English *to correctly predict* appears to have some additional reading as compared to German *korrekt vorhersagen*. The two verbs essentially have the same profile, except for a large group of participants who have a SE interpretation for English *to correctly predict*, which is entirely lacking for German *korrekt vorhersagen*. Moreover, English *to know* and German *wissen* receive fairly similar distributions, with German exhibiting somewhat more variability. We take this to suggest that the processes underlying the decision in our experiment for *wissen, to know*, and *to correctly predict* involve some sort of ambiguity either in terms of a lexical ambiguity or in terms of diverging strategies for pragmatic resolution. Finally, the very fact that this does not appear to be the case for *korrekt vorhersagen* suggests that the sort of ambiguity encountered in the other three cases is unlikely to be a pure artifact of the experimental design.

Still, we can draw only very cautious conclusions in light of the specifics of the design and data analysis. For example, the fact that in all trials we used the same payoffs will invariably limit the range of possible strategies people can adopt in the experiment, and the priors used impose a natural limit on what counts as an optimal strategy in each model. For example, if it is reasonable to make a choice of action in some condition when the subjective probability of IE is lower than 0.33, given our priors the model will assign the highest likelihood to 0.33 and not to say, 0.15—which would also explain that particular decision. Hence, to a certain extent, the fact that we do not get dots in the plots in Figure 6 all over the space is explained by the experimental design. But the frequency of the existing dots remains interpretable nevertheless.

## 4. General discussion

In the above sections, we found the SE reading to be the preferred interpretation of questions embedded under *to know* and *wissen* while the IE reading was attested to, both for English and for German. We further found that the results for *wissen, to know* and *to correctly predict* are best explained by a model that assumes that different people have different probabilities for the readings, which indicates an ambiguity between the SE and the IE interpretation, still with clear dominance for SE readings in all cases, though to different degrees. Thereby, we did not replicate the results of previous experiments in Cremers and Chemla (2016) and Cremers et al. (2017), in which the IE readings came out as the dominant interpretation of questions embedded under *know/savoir*.

For German *korrekt vorhersagen*, IE was by far the preferred interpretation. The model that showed the best fit for *korrekt vorhersagen* was the model that assumes a common distribution for the exhaustive readings, which indicates that there is no SE/IE ambiguity for this verb in German. In contrast, for English *to correctly predict* we found evidence for such an ambiguity with a preference for the SE reading. These results differ from the previous experimental results on *to predict* by Cremers and Chemla (2016). In their experiment, the IE reading was preferred. Finally, we found that for both verbs and both languages, the WE reading was hardly of any communicative relevance.

### 4.1. Lexical differences between German and English

Our experiments revealed differences in exhaustive interpretations between German and English. In this sub-section, we will take a closer look at the possible reasons for these cross-linguistic differences. Except for chance, which can be viewed as a very unlikely account for our results, there are two main possible explanations for the observed difference.

The first one we deem not very likely but should nevertheless be addressed first. As described above, the English and the German experiments were not entirely identical. In particular, in the English experiment the persons of whom the speaker was uncertain (as pertaining to the IE reading) were mentioned individually while in German they were mentioned only as possible “others.” Moreover, in English some of the fillers were triggers of scalar implicatures. To be clear, we do not see any serious possibility for the scalar implicatures in the fillers to explain the different observed patterns. This is because we do not see such differences with *to know*, even though the fillers should have affected both verbs if any. The same goes for the online (English) vs. lab (German) setting. Still, in order to rule out that the difference could have come from some unlucky combination of individually naming the persons the speaker is uncertain about in English and the existence of scalar implicatures in the fillers, we conducted a follow-up study on German in which we gathered more data under those conditions in which the English IE-data were gathered. Like in the English version of the experiment, the attitude holder explicitly named those candidates they were uncertain about in the follow-up study, and, like in the English version, the experiment was conducted in combination with an experiment on scalar implicatures, which is presented in Cortez Espinoza and Fricke (2023). The details of the follow-up experiment are explained in the Supplementary material. What matters for our purposes is that the results pattern matched exactly with the German data from the main experiment presented above.

For an inferential statistical analysis, we created a data set which included the data from the IE conditions of the verb *korrekt vorhersagen*/*to correctly predict* from the three experiments and implemented two Bayesian linear mixed models using the package rstanarm (Goodrich et al., 2022), which had, beside negation and role, experiment as a fixed factor. The model specification is shown in (15). We used default priors, which are weakly informative.

(15) stan glmer[response ~ negation + role + experiment + (1|participant) + (1|item), family = binomial, iter = 45000]

The first model differentiated between all three experiments, Experiment 1 (German), Experiment 2 (English), and the follow-up study on German. The second model considered the two German experiments together. Model comparison using Bayes Factors yielded strong evidence that the second model is superior (BF = 10.48). This supports our assumption that methodological differences between Experiment 1 and Experiment 2 are not the reason for the different prevalence of the IE reading between German and English.

The second option that could explain the cross-linguistic difference observed is that they follow from differences in the lexical semantics or in the typical uses of these verbs in German vs. English. To this end, we compared their dictionary entries and word sketches (collocations) in the two languages.^13^

According to the lexical entries from DWDS (16) and OED (17), both *vorhersagen* and *to predict* share the meaning component “state something about the future.” However, whereas for German this statement can be based on knowledge, conjecture or premonition, the entry on English *to predict* only mentions knowledge and reasoning. The OED further states that *to predict* occurs in the context of scientific law. These entries indicate that accuracy plays a greater role for *to predict* than for *vorhersagen*.

(16) über Künftiges etw. aufgrund der Kenntnis von Zusammenhngen oder aufgrund einer Vermutung, Ahnung aussagen, etw. im Voraus ankündigen
  “state something about the future based on knowlegde of context or based on conjecture, premonition”
  “vorhersagen”, in: DWDS—Digitales Wörterbuch der deutschen Sprache, ed. by Berlin-Brandenburgischen Akademie der Wissenschaften, www.dwds.de/wb/vorhersagen. Accessed 7 January 2022.
(17) (1a) To state or estimate, esp. on the basis of knowledge or reasoning, that (an action, event, etc.) will happen in the future or will be a consequence of something; to forecast, foretell, prophesy. Also, with clause as object.
  (1b) Of a theory, observation, scientific law, etc.: to have as a deducible or inferable consequence; to imply.(2) To make a prediction or predictions, to prophesy
  (3) To direct fire with the aid of a predictor“predict, v.” OED Online, Oxford University Press, December 2021, www.oed.com/view/Entry/149856. Accessed 7 January 2022.

A comparison of word sketches created with Sketch Engine^14^ (Kilgarriff et al., 2014) confirms this initial impression. We used the corpora “English Web 2018” (comprising 21,926,740,748 words) and “German Web 2018” (comprising 5,346,041,196 words). Word sketches show which words typically collocate with the word in question. Table 8 lists the first ten collocations ranked by score for *to predict* and *vorhersagen*, where the score indicates the strength of the collocation. The count represents how often a given word collocates with the verb and its nominalizations in the corpus. The ranking considers all grammatical relations together. Therefore, the noun *Fußball* occurs twice in the table, for instance, once as an accusative object, and once as a subject.

Strikingly, the 10 strongest collocates do not overlap at all for the two verbs. For *to predict*, the strongest collocate is the adjective *accurately*. Other adjectives in the top 10 are *correctly* and *reliably*. Moreover, there are three collocates from the lexical field of science: *analyst, expert* and *model*. In contrast, the 10 strongest collocates of *vorhersagen* concern soccer or gambling. There is thus a clear contrast between the typical uses of *predict* and *vorhersagen*, which relates to our experimental findings. English *to predict* appears to be associated with accuracy and science, with little tolerance for error, whereas German *vorhersagen* typically occurs in contexts in which predicting amounts to guessing. This serves as a good explanation for why the IE reading, which allows for some uncertainty on the part of the attitude holder, is more popular among speakers of German than among speakers of English.

**Table 8:** First 10 entries of word sketches: *to predict* vs. *vorhersagen*.

***To predict***			***Vorhersagen***		
**Word**	**Count**	**Score**	**Word**	**Count**	**Score**
Accurately	7,128	8.2	Bundesliga	226	7.0
Outcome	8,972	7.3	Bundesliga	586	6.8
Analyst	3,527	6.9	Tipps “predictions”	129	6.5
Correctly	2,722	6.6	Fussball “soccer”	93	6.2
Future	8,071	6.4	Sportwette “sports bet”	156	6.1
Behavior	4,471	6.3	App	66	5.7
Forecaster	1,163	6.0	Wette “bet”	137	5.5
Expert	4,315	5.9	Fussball “soccer”	56	5.4
Model	8,338	5.8	Fussball-Spiele “soccer games”	51	5.4
Reliably	1,041	5.7	Lottozahl “lottery number”	52	5.4

The comparison between *wissen* and *to know* is less informative. Examples (18) and (19) show their respective lexical entries in DWDS and OED. The entry on *wissen* represents the verb as a state or result and mentions causes for knowledge, whereas the entry on *to know* represents it as both a state and a process. It is not obvious that such differences would pertain to differences in exhaustivity, which is reflected by our quantitative finding that the differences between the two verbs in the two languages are minor.

(18) Etw. infolge eigener Erfahrung, Wahrnehmung, durch Lernen, Studium, durch Mitteilung von anderen im Gedchtnis, Bewusstsein haben und wiedergeben können, von etw. Kenntnis haben, über etw. unterrichtet sein.
  “To be aware of sth, to have sth. in mind and to be able to reproduce it, to be informed about sth. as a result of learning, studies, through communication by others.”
  <etw. zu tun wissen> etw. zu tun verstehen, imstande sein, etw. zu tun, etw. tun können
  “ <know to do sth.> To be able to do something, to be capable of doing something”
  “wissen”, in: DWDS—Digitales Wörterbuch der deutschen Sprache, ed. by Berlin-Brandenburgischen Akademie der Wissenschaften, https://www.dwds.de/wb/wissen. Accessed 7 January 2022.
(19) (1) To recognize, acknowledge, perceive. (2) To be acquainted with, have experience of. To (come to) apprehend, be or become conversant with or aware of; to learn.
  “know, v.” OED Online, Oxford University Press, December 2021, www.oed.com/view/Entry/104157?. Accessed 7 January 2022.

Using the same corpora as for *to predict* and *vorhersagen*, we created word sketches for *to know* and *wissen* as well. A ranking that combines all grammatical relations proved to be hardly informative as it contained many “semantically weak” words such as pronouns and discourse particles. Therefore, we present a comparison of the ranking of the strongest 10 collocates that are (accusative) objects in Table 9. The two verbs have an intersection of four words (*name, truth, answer, thing*). It should be noted that *someone* and *people* would not be possible collocates of German *wissen*, since it cannot be used with animate objects in the sense of “to be acquainted with.” When investigating the remaining four non-overlapping words, we do not see any hint at a difference in use that would predict different exhaustive interpretations of questions embedded under the two verbs.

We conclude that there is a difference in lexical meaning between *vorhersagen* and *to predict*, which shows both in dictionary entries and in their collocations. This cross-linguistic difference in lexical meaning is a very likely reason for the different prevalences of IE interpretations for questions embedded under these verbs in the two languages. *Wissen* and *to know*, in contrast, did not show such striking differences, so that possible minor differences in exhaustive interpretation of questions under these verbs cannot be based on differences in lexical meaning.

**Table 9:** First 10 entries of word sketches: *to know* vs. *wissen* ([accusative] objects only).

***To know***			***Wissen***		
**Word**	**Count**	**Score**	**Word**	**Count**	**Score**
Nothing	1,17,409	8.8	Bescheid^a^	20,602	10.7
Anything	90,295	8.4	Rat “advice”	5,620	8.7
Name	63,315	7.8	Antwort “answer”	5,068	7.8
Truth	48,426	7.8	Verwendung “usage”	2,626	7.4
People	90,849	7.8	Name “name”	3,397	7
Someone	49,041	7.7	Ding “thing”	2,418	6.6
Something	68,744	7.7	Genaue(res) “precise details”	918	6.5
Everything	51,292	7.7	Wahrheit “truth”	1,081	6.3
Answer	44,281	7.6	Genauer(es) “precise details”	749	6.2
Thing	75,287	7.6	....	949	6.2

^a^*Bescheid wissen* is a fixed expression that can be translated as “to be in the know,” “to be up to date,” or in many cases simply as “to know”.

### 4.2. Methodological discussion

In this section, we discuss three aspects. First, the important conclusions regarding our methodology, second, a possible explanation for our puzzle regarding the divergence between expert intuition and experimental findings and third, the discussion of the experimental methodology regarding its robustness.

Regarding the first issue: While previous experiments on the exhaustivity of embedded questions employed classical truth-value judgment and acceptability tasks, which leave open on what grounds participants' judgments are formed, our design involved a critical novel feature: participants had to consider whether a specific interpretation in question would be shared by another language user as this comparison served to maximize their financial payoff. We thereby aimed to find out which exhaustive interpretations are of communicative relevance. Of course, our experiment does not represent natural communication as such, but we regard its design as an improvement over previous experiments on this topic since it controls directly for participants' decision strategy: it is only rational to accept a target sentence in a given exhaustivity context if one is relatively certain that another person would agree with that judgment, which raises the stakes for accepting a given reading. To gauge which impact the financial aspect has, the experiment should be repeated comparing the effects of different amounts of money and the way in which the payoffs are phrased. It is conceivable that changing gains and losses in the experiment will affect participant behavior in terms of prospect theory (Kahneman and Tversky, 1979).

An additional effect of our method has been pointed out to us by a reviewer. They suggest that the task of choosing an interpretation which other people would share might have introduced a bias to choose the most restrictive interpretation. If this was the only effect of our method, we would expect that varying payoffs would not affect the participant's behavior in a repetition study. Moreover, assuming this bias, the cross-linguistic difference found between *to correctly predict* and *korrekt vorhersagen* would appear particularly robust because it came through in spite of an experimentally induced bias to choose the most restrictive interpretation.

Another important finding pertains directly to the main hypothesis of this paper: Our data provide a plausible explanation for why trained linguists would consider the SE reading the only available reading for questions under *to know*: SE is the most likely reading. Whereas the IE reading was shown to be available as well, this reading seems to come with a significantly lower probability than SE. Arguably, such lower probability readings did not find sufficient attention prior to the experimental findings reported in the literature. At the same time, our data are also in line with the previous experimental findings: IE readings still have a sufficiently high probability to account for the fact that they were robustly attested in a number of acceptability or truth-value task experiments. Finally, our experiment reveals that there is a strong prevalence of the IE reading for German *korrekt vorhersagen*. That this reading was not reported before Spector (2005, 2006) might simply follow from the fact that the difference between IE and WE readings was not sufficiently clear in earlier literature.

Finally, let us turn to the question of how robust our methodology is. To some extent the reported follow-up experiment on IE-readings of *korrekt vorhersagen* already suggests that the result do not depend very much on slight differences in wording. As expected, the financial incentive appears to be the important driving factor. But we have, in order to double-check robustness, conducted one more experiment^15^ with the same method, on German *wissen*. The details of the experiment are presented in the Supplementary material. Crucially, in that experiment we had differences in methodology. Besides some smaller differences, the presentation of the facts in the world differed from the other experiments. Instead of presenting them in the form of a table on the backside of the betting slip, a statement relating the facts was placed below the statement of the attitude holder. In spite of these differences, the results are basically the same as in the experiment presented above. In Bayesian models, the SE reading comes out dominant again and the IE reading is more readily available than the WE reading. This, again, suggests that the experimental methodology is fairly robust even against strong variations in procedure and method.

### 4.3. Theoretical discussion

Finally, we turn to the question of how our results map to the existing theoretical literature. Of course, this literature was not decidedly about the optimal readings associated with these verbs and embedded questions in a communicative setting, but rather about their underlying semantic interpretations. Still, it is an interesting question whether any of the existing theories can account for our results.

For comparison with the predictions in the literature, Table 1 from the Introduction is repeated in Table 10. The rows corresponding with our results are highlighted. A theory of question meaning needs to explain why both SE and IE interpretations exist, on the one hand, and why, on the other hand, they differ in acceptability depending on the exhaustivity properties of different embedding verbs. Uegaki (2015), Theiler et al. (2018), and Zimmermann et al. (2022) offer promising proposals in this direction.

**Table 10:** Literature overview: questions embedded under *to know*.

**Literature on *to know***	**SE**	**IE**	**WE**
Karttunen (1977), Berman (1991) Sharvit (2002), Guerzoni and Sharvit (2007), and Spector and Egré (2007)	✓	✓	✓
Groenendijk and Stokhof (1984), Heim (1994), Beck and Rullmann (1999), Lahiri (2002), George (2011), Klinedinst and Rothschild (2011), and Theiler (2014)	✓	*	*
Spector (2006), Nicolae (2013), Spector and Egré (2015), Uegaki (2015), Theiler et al. (2018), and Zimmermann et al. (2022)	✓	✓	*
**Literature on** ***to predict***	**SE**	**IE**	**WE**
Karttunen (1977), Berman (1991), Heim (1994), Beck and Rullmann (1999), Sharvit (2002), and Klinedinst and Rothschild (2011)	✓	✓	✓
Groenendijk and Stokhof (1984)	✓	*	*
Spector (2006), Theiler (2014), Spector and Egré (2015), Uegaki (2015), and Theiler et al. (2018)	✓	✓	*

Uegaki (2015) takes IE readings to be the underlying semantic interpretation of embedded questions and analyzes SE readings as pragmatic inferences: SE-readings follow pragmatically from the so-called “excluded-middle assumption.” For a sentence like *Ali knows who danced*, the assumption would be that Ali's mental state (= knowledge) applies to each relevant person; cf. Zimmermann et al. (2022) for a critical assessment.

Theiler et al. (2018) propose an inquisitive semantics analysis and assume two semantic types of question meaning, exhaustive and non-exhaustive. In combination with a complete/incomplete operator, these yield the different exhaustive readings. They further observe that knowledge ascriptions from the first-person perspective are always SE, whereas they can be IE from the third-person perspective. Based on this, they propose two lexical entries for *to know*. Given this, one could argue that in some sense SE is the most natural reading for *to know*, as observed in our experiment.

Zimmermann et al. (2022) build on these proposals. With Uegaki (2015), the authors assume that SE readings are pragmatic inferences, but they derive them from a different principle, the “Principle of Attitude Verification.” This principle is informally grounded in theory-of-mind approaches, and it rests on the idea that the most reliable evidence for somebody's knowledge state (or other mental states) is a verbal commitment of that person to this knowledge state. The principle states that in order to truthfully state of Ali that he knows who danced (in an IE- and or a SE-context), one normally assumes that Ali would commit himself to this knowledge state by saying *I know who danced*. However, this first-person report by Ali is only felicitous in an SE context, i.e., when Ali knows who danced and knows that this is the full answer; cf. Heim (1994). On this line of reasoning, though pragmatic in nature, SE would constitute the default interpretation of questions embedded under *to know*, and this is exactly what we found in our experiments. Zimmermann et al. (2022) remain silent on *to predict*, however.

Other than this, our data do not put us in a position to make any more specific claims about the semantic or pragmatic analysis of embedded questions under “to know” and “to predict.” We expect that future experimental research using a variant of our methodology could be fruitfully employed to disentangle the pragmatic and semantic aspects of the two readings, in particular, by providing a clearer picture of the probability distribution, by considering varying payoffs, and by providing more elaborate scenarios in which participants modulate their statements and their interpretations given more dynamic pragmatic goals (such as solving joint decision problems).

## 5. Conclusion

This paper investigated the pragmatic likelihood of the different exhaustive interpretations for embedded questions under the verbs “to know” and “to correctly predict” in a cross-linguistic comparison between German and English. In order to find out which interpretations are of (most) communicative relevance, we employed a decision-theoretic experimental design, in which participants had to consider the perspective of another language user to maximize their expected utility. Probabilistic models showed that for *wissen*/*to know* the SE reading had the highest probability in both languages. The IE reading was attested too but not to the extent as in earlier experiments on English and French. While in the case of *korrekt vorhersagen*, the IE reading had clearly the highest probability, *to correctly predict* behaved similarly to German *wissen*. These two verbs showed an ambiguity between the SE and the IE reading. The cross-linguistic difference between *korrekt vorhersagen* and *to correctly predict* was reflected in dictionary entries and in word sketches representing collocations of *vorhersagen* and *to predict*, which made us conclude that their difference in exhaustivity is based on a difference in lexical meaning. Finally, we discussed that Uegaki (2015), Theiler et al. (2018), and Zimmermann et al. (2022) present theories on the semantics of question which offer explanations for the observed co-existence of SE and IE readings.

## Data availability statement

The original contributions presented in the study are included in the article/Supplementary material, further inquiries can be directed to the corresponding author.

## Ethics statement

The studies involving humans were approved by Ethics Committee of the University of Graz. The studies were conducted in accordance with the local legislation and institutional requirements. The participants provided their written informed consent to participate in this study.

## Author contributions

LF and EO designed the experiment. LF created the experimental material, conducted the German experiments, performed descriptive data analysis, ran Bayesian generalized linear mixed models, and conducted the corpus study. ED conducted the English experiment. EO designed further data analysis strategy and created probabilistic Bayesian models. ED, LF, EO, and MZ wrote the manuscript. EO and MZ supervised the project. All authors are responsible for the content of the report. All authors contributed to the article and approved the submitted version.

## Footnotes

^1^In addition to the three exhaustive readings, there are also non-exhaustive (NE) interpretations or so-called mention-some readings. Such readings are typical for sentences containing existential modals or for sentences occurring in particular (non-exhaustive) contexts. For instance, the sentence *Kim knows who can lend me a drill*. is generally considered true in a situation in which Kim knows one person who would be willing or able to lend out their drill. In this paper, we will not be further concerned with mention-some readings, see Xiang (2016), Phillips and George (2018), or Moyer (2020) for some recent research on this topic.

^2^The discussion of question exhaustivity has always been central to their formal analysis (see already Hamblin, 1958 vs. Hamblin, 1973). Thus, the observation in Groenendijk and Stokhof (1984) that *to know* gives SE with embedded questions was an important discovery in view of the hazy intuitions that had been previously reported for matrix *wh*-questions. In fact, the observation of SE-readings with *to know* was the key motivation for partition semantics, one of the dominant theories of question semantics.

^3^Cremers and Chemla (2017) comment that the task employed in Cremers and Chemla (2017) leads to more SE responses but do not discuss possible reasons. There was a difference between the tenses of the target sentences in the two experiments. In Cremers and Chemla (2016) it was in the past tense and in Cremers and Chemla (2017) the target sentence was in the present tense. However, we do not have a clear hypothesis on how this might have caused the difference in the results.

^4^We thank an anonymous reviewer for pointing out this literature.

^5^Of course, utility theory has received much criticism, e.g., Tversky (1975) and Kahneman and Tversky (1979), and we readily accept that our analysis may not be the final word on this matter. However, the situation that participants faced in our experiment can be considered as relatively easy with a very simple translation between decisions and financial benefits. Moreover, we introduced some level of control regarding the effect of bias in their decisions. We found no such effect.

^6^The experimental research presented in this article was approved by the ethics committee of the University of Graz. The reference number of the approval is GZ. 39/39/63 ex 2018/19. Informed consent to participate in the study was obtained from participants.

^7^In economics, it is common practice to use such performance-based compensations. See in particular Camerer and Hogarth (1999) on the effort increasing potential of money.

^8^The full version of the experiment actually included four verbs, but in this paper, we focus on only two.

^9^Chemla and George (2016) also discuss this issue.

^10^An alternative way to think of the decision process would be to use prospect theory (Kahneman and Tversky, 1979) or cumulative prospect theory (Tversky and Kahneman, 1992). However, this step would be justified in our view if the factor role did indeed influence the decision process in any detectable way. Since, however, this was not the case, we see no reason to complicate the linking theory in this paper.

^11^We have also compared prior predictive check with posterior predictive check making sure that the quality of the simulated data improved significantly over the priors, even though the priors themselves provide fairly useful predictions.

^12^Of course, the posteriors for the three belief-values of each individual in the experiment were normally distributed, but we are discussing population level here.

^13^We compared *to predict* and *vorhersagen* without the addition of *correctly*/*korrekt* because due to the principle of compositionality semantic differences between the two expressions: “correctly predict” and “korrekt vorhersagen” should follow from the observed differences between the verbs themselves. Of course, there is still a chance that one of the complex expressions is a construction (and not compositional) but if so, this could be shown by future research.

^14^ http://www.sketchengine.eu

^15^Temporarily, the experiment we mention now was conducted first but the order of the experiments does not appear to matter.
