# Supplementary material for: The pragmatics of exhaustivity in embedded questions: an experimental comparison of know and predict in German and English
Source: Front Psychol. 2023 Sep 13;14:1148275. doi: 10.3389/fpsyg.2023.1148275 (PMC10525336; doi:10.3389/fpsyg.2023.1148275)

R2

Short description

In this linguistic experiment you will evaluate the outcome of 50 bets in the context of a fictional situation. The experiment will take 30 to 40 minutes. There will be a short break in between. Your compensation is performance-based, but in any case, you will earn a minimum of $5. You will receive your payment in the form of an Amazon gift card via email within a few days.

Declaration of consent

I consent to the processing of my answers for the scientific research at the [removed for review], the [removed for review] and the [removed for review]. Responsible: [removed for review]. The ethics committee in charge is the ethics committee of the [removed for review]. Personal data will only be used for processing the payment of the compensation. I have the right to quit the experiment at any time.

General introduction

Tiffany and Tim are the hosts of the TV reality show *The Glass House*. This show follows five contestants, Alicia, Freddy, Carlos, Mary and Sophia, who are filmed during their month long stay together in a house. Every now and again they must do certain tasks and dares. Due to their jobs, Tiffany and Tim get to know the participants very well and are well informed about the happenings on the show.

At the end of the season there is a special episode during which Tiffany and Tim talk about the show, take a look back at certain events and get asked questions about the participants and incidents on the show. Earlier, fans of the show had the possibility of placing bets on Tiffany and Tim's responses and on what activities they expected the participants to do.

Your task

You work at a betting shop and it is your job to revise the 50 betting slips that are cashed in by a person named Lilly. For each betting slip, you need to check whether Lilly won the bet or not. In addition to the contents of the bet, you will have further information on both sides of each betting slip in order to help you judge whether or not the bet was won. For each bet that Lilly won you have to pay winnings of 20 cents. If Lilly lost the bet, you do not have to pay any winnings. However, if you do not pay winnings for a bet that Lilly won, she will contest your decision and the 20 cents will be charged afterwards as well as a handling fee of 10 cents (meaning you make a loss of 30 cents rather than 20 cents).

You will now look at each of the 50 betting slips one after the other and need to decide whether the bet was won or lost. You may only look at each betting slip once and cannot change the decision you made. Each time you cash in a betting slip, there will be a display of how much money remains of your starting capital. After you revised the first half of the betting slips, you will take a short break. At the end of the experiment, you will be asked a couple of questions.

Your starting capital is $15. This is what you use to pay the winnings. Each time you cash in a betting slip, there will be a display of how much money remains of your starting capital. The amount left over at the end is what you get to keep – given Lilly doesn't contest your decisions.

An overview of the most important points:

- Profit distribution for a won bet: 20 cents
- Deduction for wrongly judging a won bet: 30 cents
- The compensation for taking part in the experiment is the amount that is left over at the end with potential deductions due to contestations. (At the very least you will get $5 for your effort.)

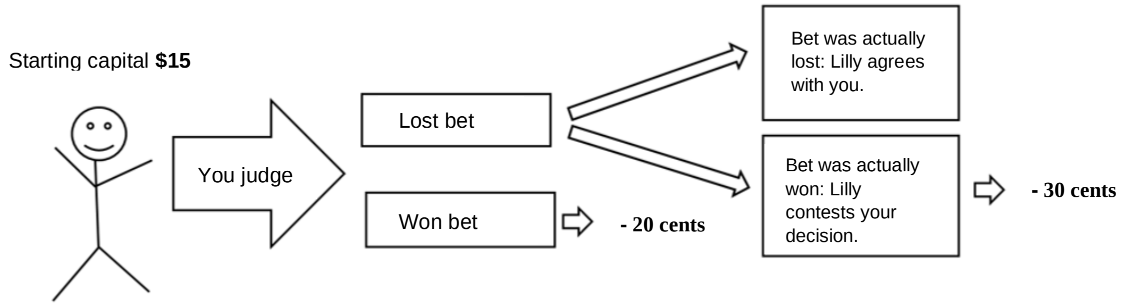

Supplement: Supplementary file 3 [file Data_Sheet_3.ZIP › Materials/Instructions_for_participants/Instructions_role_2.pdf]
