# Supplementary material for: The pragmatics of exhaustivity in embedded questions: an experimental comparison of know and predict in German and English
Source: Front Psychol. 2023 Sep 13;14:1148275. doi: 10.3389/fpsyg.2023.1148275 (PMC10525336; doi:10.3389/fpsyg.2023.1148275)

R1

Short description

In this linguistic experiment you will evaluate the outcome of 50 bets in the context of a fictional situation. The experiment will take 30 to 40 minutes. There will be a short break in between. Your compensation is performance-based, but in any case, you will earn a minimum of $5. You will receive your payment in the form of an Amazon gift card via email within a few days.

Declaration of consent

I consent to the processing of my answers for the scientific research at the [removed for review], the [removed for review] and the [removed for review]. Responsible: [removed for review]. The ethics committee in charge is the ethics committee of the [removed for review]. Personal data will only be used for processing the payment of the compensation. I have the right to quit the experiment at any time.

General introduction

Tiffany and Tim are the hosts of the TV reality show *The Glass House*. This show follows five contestants, Alicia, Freddy, Carlos, Mary and Sophia, who are filmed during their month-long stay together in a house. Every now and again they must do certain tasks and dares. Due to their jobs, Tiffany and Tim get to know the participants very well and are well informed about the happenings on the show.

At the end of the season there is a special episode during which Tiffany and Tim talk about the show, take a look back at certain events and get asked questions about the participants and incidents on the show. Earlier, fans of the show had the possibility of placing bets on Tiffany and Tim's responses and on what activities they expected the participants to do.

Your task

Your friend Lilly placed a bet on every one of the 50 questions that were asked during the special episode. However, after the special episode has aired, she does not have the time to go to the betting shop to pick up her winnings, so she asks you to do it for her. As a small thank you, you get to keep a part of the winnings. For every bet she won, you get 30 cents. Cashing in a betting slip costs you a handling fee of 10 cents per betting slip, however. Hence, you actually make 20 cents per bet you won. If you cash in a betting slip with a lost bet, you lose 10 cents. It is therefore important to always consider which betting slips to cash in and which ones not to cash in.

You are now going to look at each of the 50 betting slips one after the other. For each betting slip you must decide whether you would like to cash it in or not. In addition to the contents of the bet, you will see further information on both sides of the betting slips in order to help you decide whether or not to cash in the betting slip. You may only look at each betting slip once and cannot change the decision you made, once you have decided whether to cash in the betting slip or not. After you revised the first half of the betting slips, you will take a short break. At the end of the experiment, you will be asked a couple of questions.

You will get $5 as a starting capital that you can use to cash in the bets. Each time you cash in a betting slip, there will be a display of how much money remains of your starting capital. Your eventual compensation for taking part in the experiment will depend on how much profit or loss you make by cashing in the betting slips. This will be evaluated after the experiment. Subsequently, you will receive your compensation after the experiment.

R1

An overview of the most important points:

- Handling fee for cashing in a betting slip: 10 cents
- Your net gain for winning a bet: 20 cents
- The compensation for taking part in the experiment depends on how much profit or loss you make by cashing in the betting slips. (At the very least you will get $5 for your effort.)

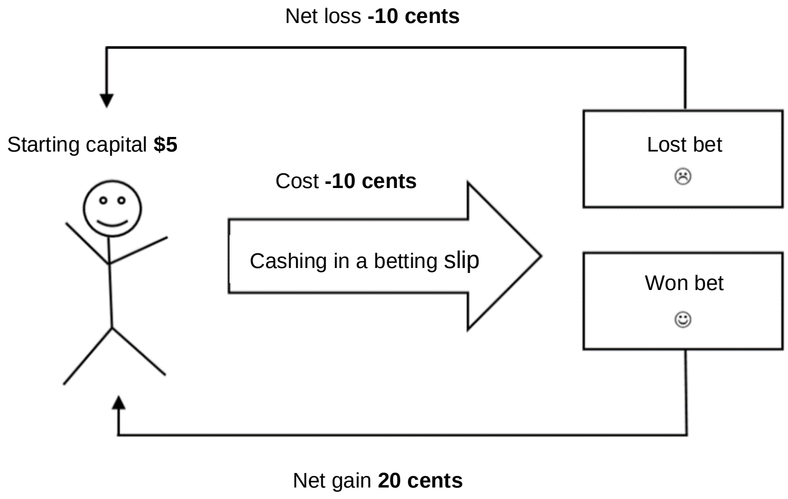

Supplement: Supplementary file 3 [file Data_Sheet_3.ZIP › Materials/Instructions_for_participants/Instructions_role_1.pdf]
