# Supplementary figures and images for: The pragmatics of exhaustivity in embedded questions: an experimental comparison of know and predict in German and English
Source: Front Psychol. 2023 Sep 13;14:1148275. doi: 10.3389/fpsyg.2023.1148275 (PMC10525336; doi:10.3389/fpsyg.2023.1148275)

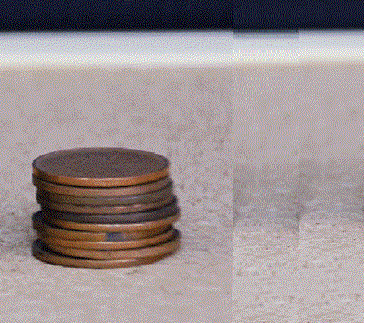

Supplement: Supplementary file 3 [file Data_Sheet_3.ZIP › Materials/Programme_to_run_experiment_online_and_stimuli/finalcoinsunder700.gif]

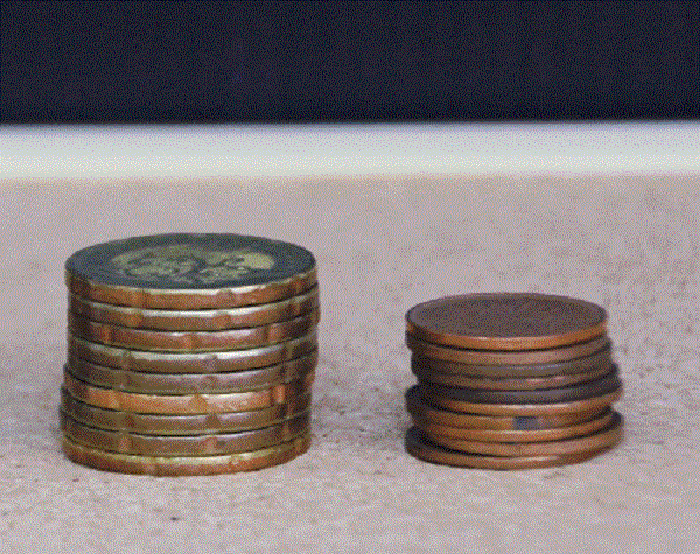

Supplement: Supplementary file 3 [file Data_Sheet_3.ZIP › Materials/Programme_to_run_experiment_online_and_stimuli/money_decreased_img2.gif]

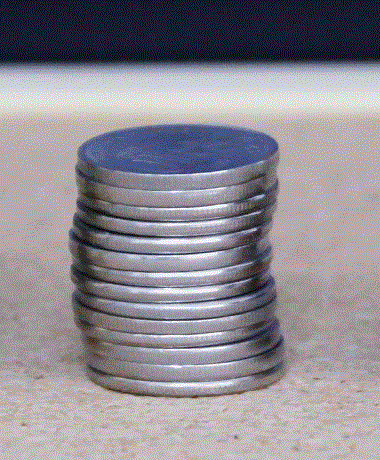

Supplement: Supplementary file 3 [file Data_Sheet_3.ZIP › Materials/Programme_to_run_experiment_online_and_stimuli/money_decreased.gif]

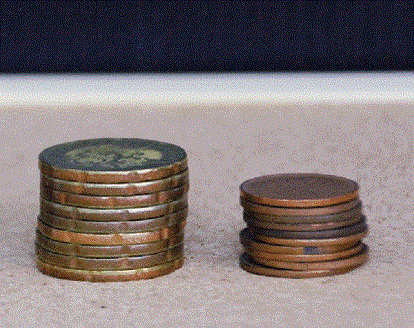

Supplement: Supplementary file 3 [file Data_Sheet_3.ZIP › Materials/Programme_to_run_experiment_online_and_stimuli/finalcoinsover700.gif]

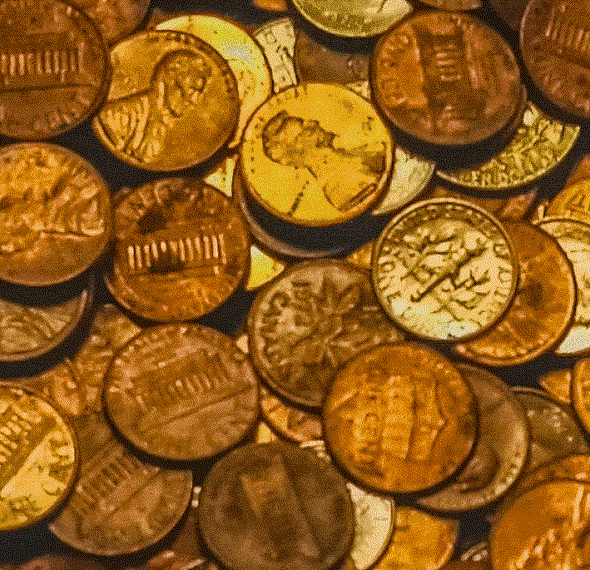

Supplement: Supplementary file 3 [file Data_Sheet_3.ZIP › Materials/Programme_to_run_experiment_online_and_stimuli/current_balance.gif]

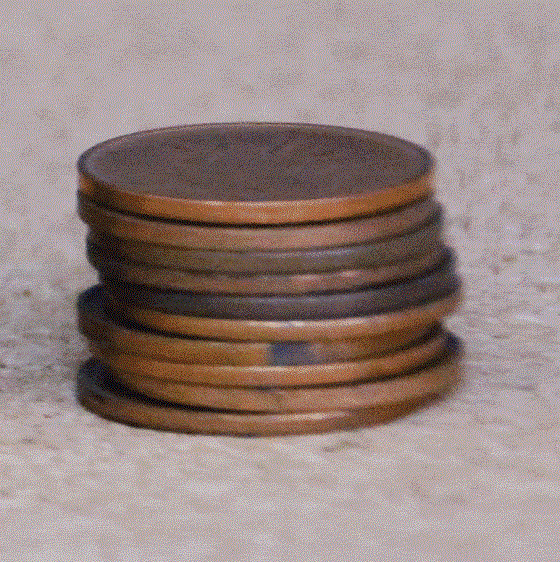

Supplement: Supplementary file 3 [file Data_Sheet_3.ZIP › Materials/Programme_to_run_experiment_online_and_stimuli/money_decreased_below300.gif]

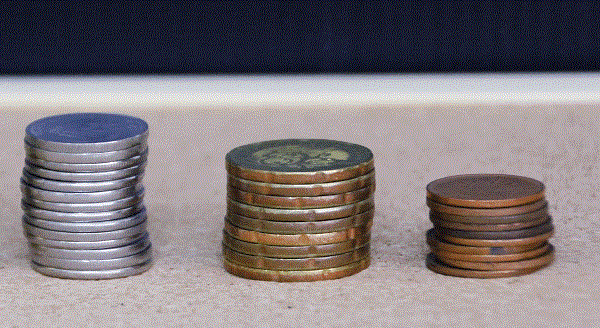

Supplement: Supplementary file 3 [file Data_Sheet_3.ZIP › Materials/Programme_to_run_experiment_online_and_stimuli/finalcoinsover1000.gif]

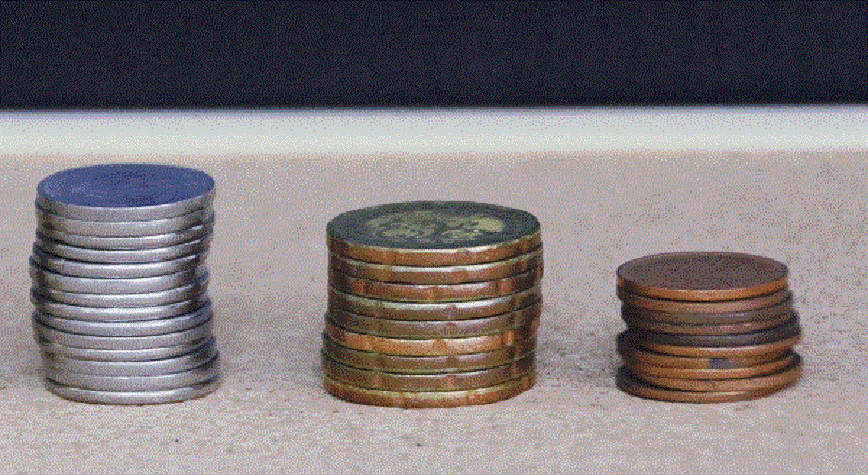

Supplement: Supplementary file 3 [file Data_Sheet_3.ZIP › Materials/Programme_to_run_experiment_online_and_stimuli/money_decreased_img1.gif]

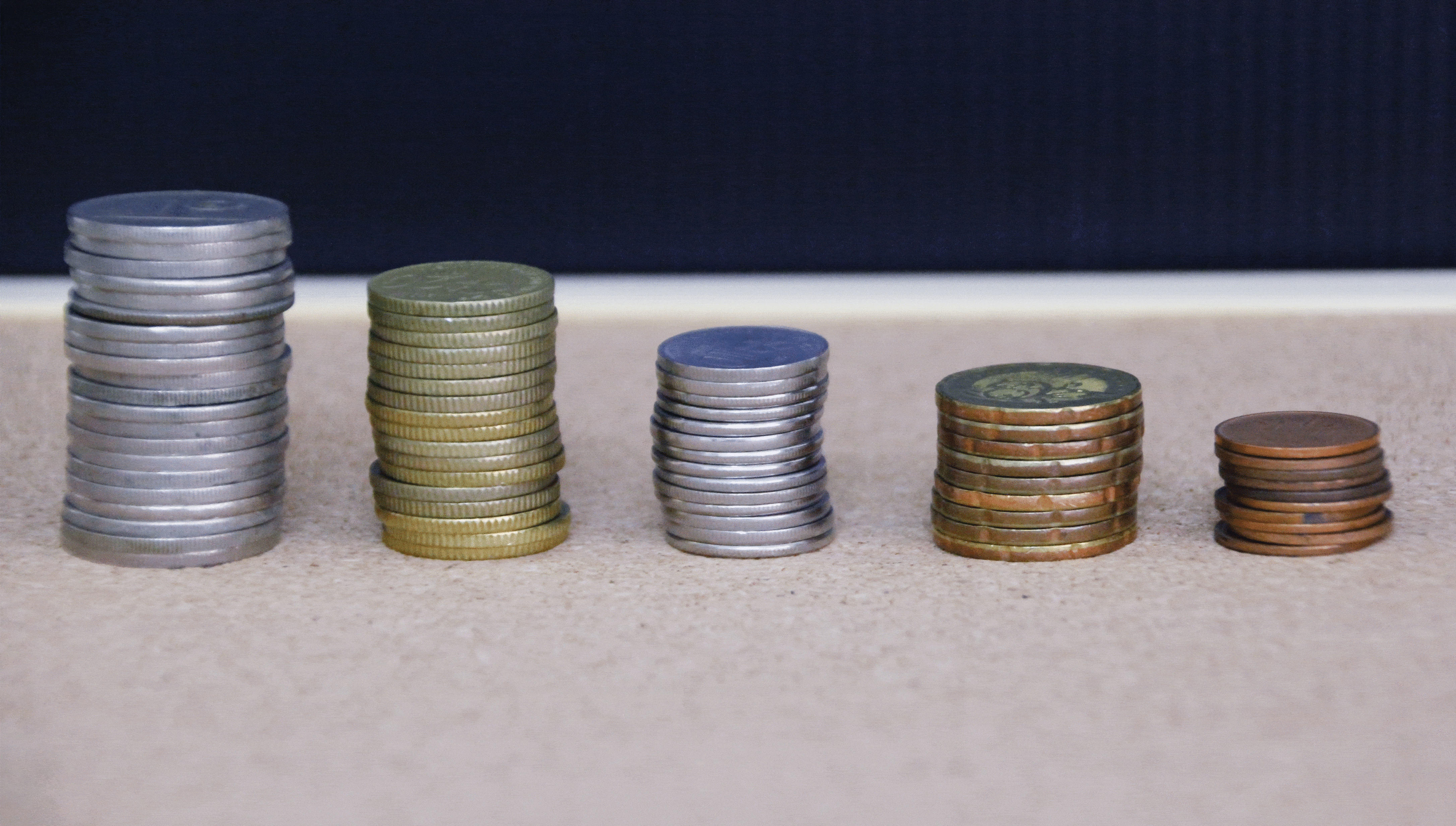

Supplement: Supplementary file 3 [file Data_Sheet_3.ZIP › Materials/Programme_to_run_experiment_online_and_stimuli/angie-j-XrhrDsZML9Q-unsplash (1).gif]
