# Supplementary material for: The pragmatics of exhaustivity in embedded questions: an experimental comparison of know and predict in German and English
Source: Front Psychol. 2023 Sep 13;14:1148275. doi: 10.3389/fpsyg.2023.1148275 (PMC10525336; doi:10.3389/fpsyg.2023.1148275)
Supplement: Supplementary file 4 [file Data_Sheet_4.PDF]

Supplementary Material

1 RHAT VALUES AND DATA DENSITY OVERLAY FOR STANDARD MODEL

Data	SE	IE	WE
wissen	1.000200	1.000189	1.000081
to know	1.000348	1.000278	1.000136
vorhersagen	1.000225	1.000051	1.000155
to predict	1.000150	1.000191	1.000290

Table S1. Rhat Values for the Standard Model simulations

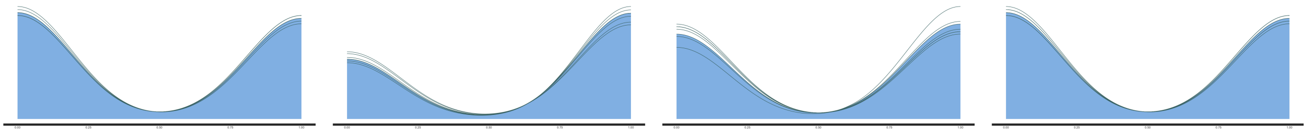

Figure S1. Predicted and actual data densities overlay for standard model: wissen, vorhersagen, to know, to predict

2 FOLLOW UP EXPERIMENT: GERMAN *KORREKT VORHERSAGEN*

This follow-up experiment retested the IE condition for German *korrekt vorhersagen* 'correctly predict' with the wording that was used in the English version of the experiment and with fillers that included scalar implicatures in order to be able to exclude the possibility that the different results of *vorhersagen* and *to predict* are due to the difference in wording or due to the presence of scalar implicatures in the fillers.

Participants
We tested 32 native speakers of German (mostly Austrian German), 15 females and 11 males, who were between 18 and 47 years old (M = 23.8), all of which were university students or former university students. They were recruited via a university-newsletter and received a financial compensation varying between 8.50€ and 11.20€.

Materials
As in the English version of the experiment, the attitude holder explicitly named those candidates they were uncertain about. The experiment was conducted in combination with an experiment on scalar implicatures. Due to the requirements of the other experiment, the context story was slightly altered to the effect that there were nine candidates in the show, instead of five, and they were all female. Besides, half of the target sentences were in past tense, as shown in (1-a), (in the previous version of the experiment, we also used past tense) and the other half was in future tense as shown in (1-b).

(1) a. Tiffany hat korrekt vorhergesagt, wer von den Teilnehmerinnen in der Sendung einen Wutanfall bekommen würde.
Tiffany correctly predicted who of the (female) participants would throw a tantrum on the show

b. Tiffany wird korrekt vorhersagen, wer von den Teilnehmerinnen in der Sendung einen Wutanfall bekommen wird.
Tiffany will correctly predict who of the (female) participants will throw a tantrum on the show.

Materials
The factorial design was 2 (NEGATION) x 2 (ROLE). We created a set of four lexicalizations, yielding a total of 8 test items. There were 16 experimental lists which included four test items and 28 fillers each, 8 of them controls on each list.
Procedure
The experiment was conducted in a lab and followed the same procedure as the main version of the German experiment.
Results
Table S2 compares the acceptances rates for each condition by role and Table S3 compares them by tense. Note that tense was not a factor in the experiment, but we wanted to check whether it affected the results. To test for effects of the factors role and tense, we fitted four Bayesian generalized linear mixed models, which we compared using Bayes Factors. The full model contained negation, role and tense as fixed factors. The reduced model contained only negation as fixed factor. The other two models contained either tense or role beside negation as a fixed factor. Model comparison showed that the reduced model is superior to each of the alternative models (Bf = 65.37 in comparison with the full model; BF = 9.08 in comparison with the model that included role; BF = 7.23 in comparison with the model that included tense). We thus conclude that neither tense nor role affected the results.

Table S2. Comparison by role: Acceptance in percent (absolute numbers in brackets)

Condition	role 1	role 2
IE no neg	83% (25)	91% (29)
IE neg	23% (7)	12% (4)

Table S3. Comparison by tense: Acceptance in percent (absolute numbers in brackets)

Condition	past	future
IE no neg	93% (28)	81% (26)
IE neg	7% (2)	28% (9)

3 EXPERIMENT ON GERMAN *WISSEN*

This experiment tested the acceptability of the three exhaustive readings (SE, IE and WE) for the German verb *wissen* ‘to know’ and for a baseline condition including false beliefs only. The baseline condition is abbreviated as NK, short for ‘no knowledge’.

Participants
We tested 24 native speakers of German (mostly Austrian German), 13 females and 11 males, who were between 18 and 55 years old (M = 24.5 years). 21 of them were university students. The financial compensation varied between 9 and 10.50 euros.
Materials
The presentation of the facts in the world differed from the other experiments. Instead of presenting them

in the form of a table on the backside of the betting slip, a statement relating the facts was placed below the statement of the attitude holder. (2) is an example item.

(2) Lina wettet:
Lina bets:
Tim weiß wer von den Teilnehmerinnen und Teilnehmern in der Sendung eine Schlange gestreichelt hat.
Tim knows who of the participants petted a snake on the show

Was Tim antwortet: “Alessa, Freddy und Carlo haben in der Sendung eine Schlange gestreichelt, aber Mara und Sophie haben in der Sendung keine Schlange gestreichelt.”
What Tim replies: “Alessa, Fredddy and Carlo petted a snake on the show, but Mara and Sophie did not pet a snake on the show.”

Was passiert ist: Alessa, Freddy und Carlo haben in der Sendung eine Schlange gestreichelt. Mara und Sophie haben in der Sendung keine Schlange gestreichelt.
What happened: Alessa, Freddy and Carlo petted a snake on the show. Mara and Sophie did not pet a snake on the show.

A further difference was that we additionally tested a condition in which the attitude holder’s beliefs were completely false. This no knowledge (NK) condition served as a negative baseline.

Factorial design

The factorial design was 4 (READING) x 2 (ROLE) x 2 (NEGATION). We tested a set of 20 test items and 30 fillers. There were 2 test item with the SE and NK reading, respectively, and 8 test items with the the IE and WE reading, respectively. We chose to test a larger number of test items with the latter readings because they constitute the readings of interest. In contrast, we assumed the acceptability of SE readings and the unacceptability of NK readings to be uncontroversial.

Procedure

The procedure did not include the three trial items at the beginning of the experiment. Otherwise the procedure was the same as in the main experiment.

Results

We excluded the data from 2 participants who made 3 or more mistakes on filler/control items. We, thus collected 44 data points in the SE and NK conditions and 176 data points in the IE and WE condition. The results are shown in Table S4. These descriptive data indicate no striking differences between the two roles. To test for an effect of the factor role, we create two Bayesian generalized linear mixed models, one of which contained the factor role beside an interaction term of the factor reading and negation and random intercepts for participants and items. Model comparison using Bayes Factors yielded strong evidence that the model which did not include the factor role was superior (BF = 17.89) to the model that included role as a factor. These results are very similar to the results of the main experiment on German. To facilitate seeing the visual similarity, we plotted the posteriors for the readings based on the standard model and the variable value model for the results of this additional experiment, excluding the NK data, in Figure S2.

Table S4. Acceptance in percent by role (absolute numbers in brackets)

Condition	Role 1	Role 2
SE no neg	100% (11)	100% (11)
SE neg	18% (2)	0%
IE no neg	45% (20)	41% (18)
IE neg	59% (26)	73% (32)
WE no neg	11% (5)	9% (4)
WE neg	93% (41)	87% (39)
NK no neg	9% (1)	0%
NK neg	100% (11)	100% (11)

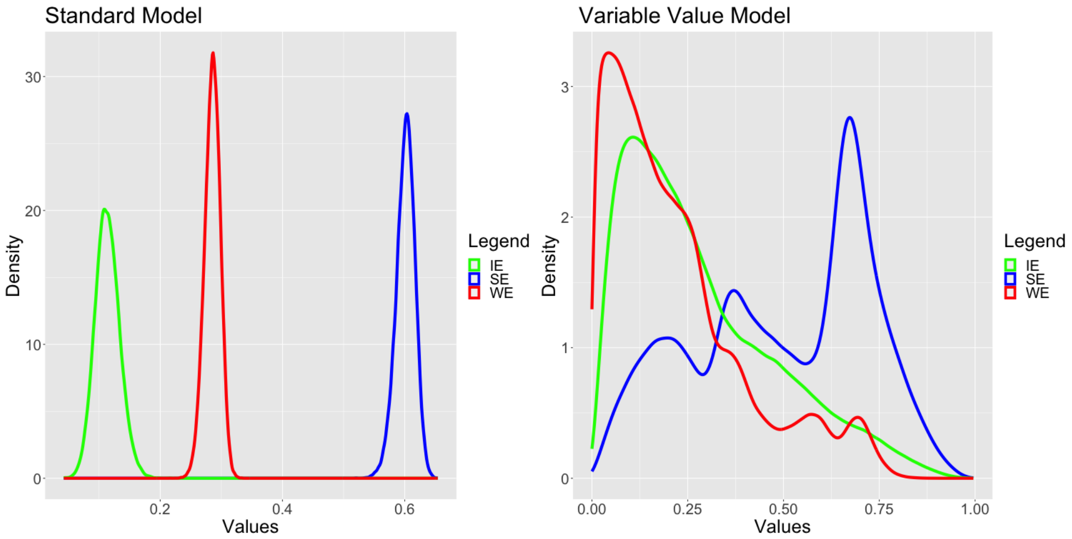

Figure S2. Posteriors for German *wissen* follow up experiment

While the results show that there is some amount of divergence at the level of the standard model, at the level of the variable value model, which is vastly superior in terms of Bayes Factor[1], the SE reading comes out dominant again and the IE reading is more readily available than the WE reading.

[1] Bayes factor of variable-value model over standard model = 3.09e+81
